# Melatonin rescues pregnant female mice and their juvenile offspring from high fat diet-induced alzheimer disease neuropathy

**DOI:** 10.1016/j.heliyon.2024.e36921

**Published:** 2024-08-24

**Authors:** Amin Jan, Mohsin Shah, Shahid Ali Shah, Syed Hamid Habib, Ehtesham Ehtesham, Naseer Ahmed

**Affiliations:** a Institute of Basic Medical Sciences, Khyber Medical University, Peshawar, Pakistan; b Department of Biochemistry, Haripur University, Haripur, Pakistan

**Keywords:** HFD, Obesity, Neuroinflammation, Neurodegeneration, Melatonin treatment, Synaptic dysfunction, ROS, Oxidative stress, SIRT1/Nrf2

## Abstract

High fat diet (HFD) is a prime factor, which contributes to the present epidemic of metabolic syndrome. Prolonged intake of HFD induces oxidative stress (OS) that in turn causes neuroinflammation, neurodegeneration, insulin resistance, amyloid burden, synaptic dysfunction and cognitive impairment hence leading to Alzheimer's disease neuropathy. Melatonin (secreted by the Pineal gland) has the potential to nullify the toxic effects of reactive oxygen species (ROS) and have been shown to ameliorate various complications induced by HFD in rodent models. This study aimed to assess the neurotherapeutic effects of melatonin on HFD-induced neuroinflammation and neurodegeneration mediated by OS in pregnant female mice and their offspring. Western blotting, immunohistochemistry and antioxidant enzyme assays were used for quantification of samples from the hippocampal region of the brain of pregnant albino mice and their offspring. Short- and long-term memory was assessed by Y-maze and Morris Water Maze tests. HFD significantly induced OS leading to AD like neuropathology in the pregnant mice and their offspring while melatonin administration simultaneously with the HFD significantly prevented this neuropathy. This study reports that melatonin exerts these effects through the stimulation of SIRT1/Nrf2/HO-1 pathway that in turn reduces the HFD-induced OS and its downstream signaling. In conclusion melatonin prevents HFD-induced multiple complications that ultimately leads to the memory dysfunction in pregnant female mice and their successive generation via activation of SIRT1/Nrf2 signaling pathway.

## 1 Introduction

Diet containing high amount of refined sugars and saturated fats are leading factors contributing to the present epidemic of metabolic syndrome [1]. High fat diet (HFD) induced metabolic disorders can have negative implication on cognition, behavior, and brain health [2]. Several human studies have shown a strong correlation with the development of Alzheimer's disease (AD) and other cognitive impairments with HFD, obesity, diabetes mellitus (DM), and other metabolic disorders [3,4]. Inflammation in various parts of the brain is linked to abnormal behavioral, cognitive dysfunction and AD [5]. Chronic HFD consumption is one of the factors that induce inflammation through the release of pro-inflammatory cytokines by adipocytes [6]. Pro-inflammatory cytokines cross the blood-brain barrier to the brain and hypothalamus, resulting in NF-κB activation in microglial cells in the hypothalamus and consequently leading to hypothalamic inflammation and leptin resistance [7]. Neuroinflammation is initially a defensive response however, excessive inflammatory reactions can be harmful and actually impede neuronal regeneration [8]. It is previously reported that diet rich in refined sugar and saturated fats detrimentally impact cognitive functions [9]. One of the major factors that exacerbates AD is the increased production of Amyloid β (Aβ) production in brain after 16 weeks HFD [10]. Furthermore, HFD accelerates the progression of AD-like pathology by increasing brain amyloid deposition and oxidative stress (OS) [11].

Increased Consumption of HFDs has been shown to induce obesity and insulin resistance (IR) [12]. Furthermore, IR could regulate Aβ production via enhancing β- and γ-secretase activity [13]. Meanwhile, IR induces oxidative stress and inflammation in the brain which contributes to Aβ and tau pathology. Aβ accumulation can enhance IR through Aβ-mediated inflammation and oxidative stress [14]. IR is a possible link between amyloid plaques and neurofibrillary tangles pathology via oxidative stress and neuroinflammation [15].

Melatonin is an important hormone secreted mainly by the pineal gland that acts as a potent anti-oxidant, anti-apoptotic and anti-inflammatory agent [16]. Melatonin also has been shown to upregulates several antioxidant enzymes and subsequently downregulate pro-oxidants, in both pre-clinical and clinical studies by modifying the pro-inflammatory and anti-inflammatory cytokines [16,17]. It also has anti-apoptotic properties mainly by inhibiting the reduction of pBad and pAkt levels [[18], [19]]. Maternal circulating melatonin levels gradually increase after 24 weeks of pregnancy spiking at 32 weeks and then reverting to baseline after delivery in a normal pregnancy [[20], [21]].

Although previous reports have shown that melatonin activate Sirtuin 1 (SIRT1) which in turn reduce oxidative stress, inflammation and neurodegeneration in various disease models by stimulating Nuclear factor E2-related factor 2/Heme-oxygenase-1 (Nrf2/HO-1) signaling pathway [[22], [23]]. However, whether melatonin will follow the same SIRT1/Nrf2/HO-1 signaling pathway to reduce the HFD-induced neuroinflammation and neurodegeneration mediated by OS in pregnant mice and their offspring is hitherto unknown. Here, we intend to see the effects of melatonin against HFD-induced changes in amyloid beta (Aβ) and synaptic markers in the dams and their offspring, providing a potential useful therapeutic index for preventing neurodegeneration and its consequences.

## 2 Materials and method

### 2.1 Study design and approval

This was an experimental study carried out at the Institute of Basic Medical Sciences (IBMS), Khyber Medical University (KMU), Peshawar, Pakistan, under Institutional Ethical Board approval number (Dir/KMUEB/EM/000806). The study consisted of three maternal and two offspring interventions, described below.

### 2.2 Animals and treatment

Healthy male (kept for mating purpose only) and female adult BALB/C albino mice (7–8 weeks of age and 30–35g of body weight) were purchased from Veterinary Research Institute, Peshawar and were kept in the animal house facility of IBMS, KMU Peshawar. Mice were housed in a climate-controlled room at 25 ± 1.5 °C, with a 12 h light and dark cycle, with access to food and water ad libitum. Animal handling was also in accordance with the European Union regulations on animal research guidelines.

### 2.3 Melatonin and special diet

Melatonin was purchased from Sigma Aldrich Chemical Co. (St Louis, MO, 63103United States) dissolved in 0.1 % dimethyl sulfoxide (DMSO) and diluted with 0.9 % saline solution. Melatonin was injected intraperitoneally, consistently between 7 p.m. and 8 p.m., at a dose of 10 mg/kg body weight. Mice that did not receive melatonin were injected with an equivalent amount of vector (0.1 % DMSO and 0.9 % saline solution). Locally prepared obesogenic diet was used as HFD, containing 45 % fats, 35 % carbohydrates, and 20 % proteins per dry weight.

### 2.4 Experimental protocol

Sixty albino mice (36 female adult mice and 24 offspring) were included in this study. Sample size was calculated via resource equation approach. The 36 adult female mice were distributed into two groups initially i.e. Control group (n = 13) fed on normal standard diet and HFD group (n = 23) was fed on HFD. After three weeks, one set (n = 3) from each group was selected randomly and assessed for weight gain and behavioral studies and then euthanized for laboratory investigations including Western blotting, Immunohistochemistry and anti-oxidant analysis. Plasma of all the euthanized mice was also analyzed for lipid parameters, i.e., Total cholesterol (TC), triglycerides (TG), high density lipoprotein (HDL), low-density lipoprotein (LDL), and very low-density lipoprotein (VLDL).

The remaining Control group (n = 10) and HFD group (n = 20) mice were allowed to mate for about one week time. After successful conception, the HFD fed mice were randomly divided further into two sub-groups (n = 10 each). The study in this stage comprises of three groups, i.e., a Control group, HFD group and a HFD + Mel group. Melatonin was administered intra-peritoneally to the HFD + Mel group pregnant mice daily till the termination of pregnancy. Equal volume of vector was administered to the Control and HFD group mice.

The HFD animals were fed HFD for 4 weeks before receiving melatonin or vehicle treatments. This protocol was selected to first establish HFD-induced changes before testing, whether melatonin can reverse or slow down the pathogenesis of obesity. Mice in the HFD and HFD + Mel groups continued HFD feeding until the termination of pregnancy. Mice (n = 5) from each group i.e. Control, HFD and HFD + Mel groups were subjected to behavioral studies and intraperitoneal glucose tolerance test (IPGTT) and then euthanized for further laboratory investigations in the 2nd maternal intervention at the termination of pregnancy. Mice (n = 3) were subjected to Western blot analysis while 2 mice were analyzed through immunohistochemistry technique. Plasma of all the euthanized mice was also analyzed for lipid parameters.

Remaining dams (n = 5/group) were allowed to complete the weaning period. After completion of the weaning period, all the 5 mice from each category i.e. Control, HFD and HFD + Mel groups were again subjected to behavioral studies, IPGTT and then euthanized for further laboratory investigations in the 3rd maternal intervention. Mice (n = 3) were subjected to Western blot analysis while 2 mice were analyzed through immunohistochemistry technique. Plasma of all the euthanized mice was also analyzed for various lipid parameters.

The 24 juvenile offspring of their respective parental dams were distributed into the same three groups i.e. Control, HFD and HFD + Mel groups with 8 offspring in each group. First offspring intervention with three pups of each respective group was carried out at the age of four weeks. Selected proteins were analyzed in the four weeks’ offspring intervention. Remaining offspring from respective parental groups (n = 5) were kept to grow to reach 8 weeks of age. These juvenile offspring were subjected to behavioral studies and IPGTT and then euthanized for further laboratory investigations. Mice (n = 3) from each group were subjected to Western blot analysis, while 2 mice were analyzed through immunohistochemistry technique in the juvenile offspring intervention. Plasma of all the euthanized mice was also analyzed for various lipid parameters.

(Project execution plan and schematic presentation of animal placement in various groups are shown in supplementary Figures S-1 and S-2 respectively).

### 2.5 Behavioural assessment

Therapeutic effects of melatonin on HFD induced memory impairment were evaluated through Y-Maze and Morris Water Maze (MWM) behavioral tests. All animals were tagged, randomized and behavioural studies were carried out as a single blinded trial. The investigator carrying out the behavioural tests was blinded to mice tags and treatment.

Y-maze spontaneous alternation test was used to measure the mice willingness to explore new environment as reported previously [24]. Y-maze test is used to evaluate short to long term spatial memory function in mice. Each time mice were allowed to get familiar with the apparatus for 10 min. Later on mice were placed in maze center, and allowed to move freely in the three arms for 8 min. Total arm entries and successive triplets of each mouse were observed and noted. The percentage of alternations was calculated by the formula as follows:

$$\text{Spontaneous}\;\text{Alternation}=\frac{\text{Succesive}\;\text{Triplet}\;\text{Sets}}{\text{Total}\;\text{Number}\;\text{of}\;\text{Arm}\;\text{Entries}\;-2}\times 100$$

Mice spatial memory was positively correlated with the percentage of spontaneous alternations.

To probe the mice hippocampus based long-term spatial learning abilities, MWM test was carried out. The design and dimensions of MWM test apparatus are specified in detail recently [25]. Before starting the tests, mice were trained for swimming twice a day for three days to acclimatize with water tank and platform. Later, the escape latency was calculated for each mouse for a period of 60 s to find the submerged platform and this was continued for 05 days. The mice were manually directed and placed on platform for 10 s if failed to find the platform in specified time period (60 s). Escape latency time was recorded for each day. Probe test was performed after two days of rest. Mean time spent in target quadrant by each mouse was calculated.

### 2.6 Intraperitoneal glucose tolerance test

Mice were kept on overnight fast (10:00 p.m.-9:00 a.m.) with free access to water. After overnight fast, their blood glucose levels were measured at the start (0 time), prior to the intraperitoneal administration of 2.5 g/kg glucose solution. The blood glucose level was then measured at different time points i.e. 15, 30, 60, 120 and 180 min respectively, with a glucometer (Accu-Chek by Roche Diabetes Care, Inc. Switzerland).

### 2.7 Biochemical analysis of plasma

Blood was collected after euthanasia by left intraventricular puncture and immediately mixed with 77 mM EDTA, then centrifuged at 3.0×*g* and 4 °C to obtain plasma. Serum was used for biochemical analysis. Semi-automatic biochemistry analyzer RT-1904C was used for measuring lipid parameters, i.e., TC, TGL, HDL, LDL, and VLDL.

### 2.8 Western blotting

All animals were euthanized at the end of each intervention after completion of behavioral studies and IPGTT for Western blotting as per method reported previously [26]. Briefly, mice were decapitated and the whole brain was carefully extracted. Hippocampus was homogenized and tissue supernatant was collected and stored at −20^o^C till further analysis. Bio-Rad protein assay solution was used to quantify the hippocampal homogenates. The homogenates were fractionated using SDS-PAGE (Model: BIO-RAD Mini PROTEAN System Cat#1658050, USA) with 10 % polyacrylamide gel. After transfer, the membranes were blocked with 5 % skimmed milk, incubated overnight at 4^o^C with primary antibodies. Different mouse derived primary antibodies such as anti-BCL-2 (SC-7382), anti-BAX (SC-7480), anti-CAS-3 (SC-7272), anti-PARP-1 (SC-8007), anti-SIRT1 (SC-74504), anti-NRF-2 (SC-365949), anti-HO-1 (SC-136960), anti-Aβ (SC-28365), anti-BACE1 (SC-33711), anti-PSD95 (SC-71933), anti-SYP (SC-17750), anti-p-IRS (SC-17196), anti-p-AKT (SC-514032), anti-p-GSK3B (SC-81496), anti-TNF-α (SC-52746), anti-COX2 (SC-376861), anti-IL-1β (SC-32294) and β-actin (SC-47778) of Santa Cruz Biotech, CA, USA were used to detect and localize protein of interest. ECL (enhanced chemiluminescence) was used to detect cross-reacting proteins after incubation with horseradish peroxidase-conjugated secondary antibodies. ECL detection reagent was used for visualization according to the manufacturer's instructions. ImageJ software was used to perform the densitometry analysis of the bands in arbitrary units (A.U.) relative to the untreated control.

### 2.9 Tissue preparation and immunohistochemistry

Equal number of mice (n = 2) were kept in each group for conducting morphological study through immunohistochemistry. Transcardial perfusion with phosphate buffered saline (PBS) and 4 % ice-cold paraformaldehyde was performed. After post-fixing in 4 % paraformaldehyde overnight, the samples were transferred to 20 % sucrose solution until the brains sank to the bottom of the tube. Brains were frozen in optimum cutting temperature (O.C.T) compound (A.O. USA) before obtaining 16 μm sections in the coronal planes using a Leica cryostat (CM 3050C; Germany). The sections were thawed and mounted on probe-on plus charged slides (Fisher). Immunochemistry staining was done as reported previously [27]. Solution of 0.01M phosphate buffered saline (PBS) was used to wash slides twice for 15 min. After washing blocking solution and proteinase K was added. Primary anti-bodies, anti-SIRT1, anti NF-κB and anti-Aβ were added and kept at 4^o^C overnight. Secondary anti-bodies (FITC and TRITC conjugated, Santa Cruz, 1:50 in PBS) were added for additional 90 min at room temperature after washing with PBS on the next morning. Slides were washed for 5 min with PBS. DAPI (4′, 6-diamidino-2-phenylindole) and PI (propodeum Iodide) were applied to stain nucleus and slides were mounted with glass cover slips with mounting medium. Images were captured with confocal microscopy (FluoView FV 1000 Olympus, Japan). ImageJ software was used to perform the densitometry analysis of the percentage area stained in each image in arbitrary units (A.U.) relative to the untreated control.

Additional details for some procedures and results are given in the Supplementary document.

### 2.10 Statistical analysis

IPGTT and Morris water maze results are analyzed through two-way analysis of variance (ANOVA), followed by Post-hoc tukey's test. Biochemical, oxidative stress markers and weight changes were analyzed using one-way analysis of variance (ANOVA), followed by Post-hoc tukey's test. The original X-ray films of the Western blot analysis were scanned. Image J software was used to perform densitometry analysis of the bands. Integral optical density of proteins were expressed in arbitrary units (A.Us) as Mean ± S.E.M. Significant difference in the first intervention was determined using *t*-test whereas significant differences in the 2nd and 3rd maternal and both offspring interventions was determined using one-way analysis of variance (ANOVA), followed by Post-hoc tukey's test. Immunohistochemistry images were also analyzed through Image J software for obtaining Integral optical density of proteins. Significant differences in the 2nd and 3rd maternal and both offspring’ interventions was determined using one-way analysis of variance (ANOVA), followed by Post-hoc tukey's test. A value of p < 0.05 was considered statistically significant. Graph Pad Prism 8 software (San Diego, CA, USA) was used for statistical analysis.

## 3 Results

### 3.1 HFD feeding induced multiple complications in female mice

**Fig. 1:**
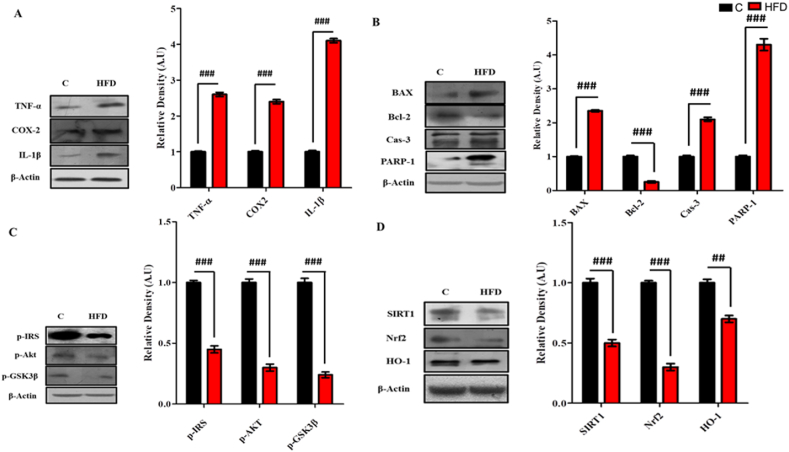
HFD induced significant changes in the expression levels of various markers in the hippocampus of the Dams. Shown are the Western blot results of maternal (A) neuroinflammatory markers (COX2, TNF-α and IL-1β), (B) neurodegeneration markers (BAX, Bcl-2, Cas-3 and PARP-1), (C) insulin resistance markers (p-IRS, p-Akt and p-GSK3β) and (D) signaling proteins (SIRT1, Nrf2 and HO-1) in pre-pregnancy stage, along with their bar-chart representation respectively. The assay was repeated three times. β-Actin was used as a loading control. The results were determined using Image J software and bar-chart indicate mean in A.U ± SEM. Significance of control vs HFD is expressed as #. Significance: ##p < 0.01 and ###p < 0.001.

A statistically significant increase (p < 0.001) in the expression of neuroinflammatory markers (TNF-α, COX2 and IL-1β) (Fig. 1A), pro-apoptotic marker, PARP-1 (p < 0.001) and significant decrease (p < 0.001) was observed in the anti-apoptotic BCl-2 in the HFD fed mice. Likewise, significant increase (p < 0.001) was observed in the Bax and Caspase-3 levels (Fig. 1B). Similarly, significant decrease (p < 0.001) in the expression of p-IRS-1, p-Akt and p-GSK3-β levels were observed in the hippocampal homogenates of HFD group as compared to normal chow diet group (Fig. 1C). Significant decrease (p < 0.001) was also observed in the expression of SIRT-1, Nrf-2 and HO-1 (p < 0.01) in HFD group as compared to normal chow diet group (Fig. 1D).

**Fig. 2:**
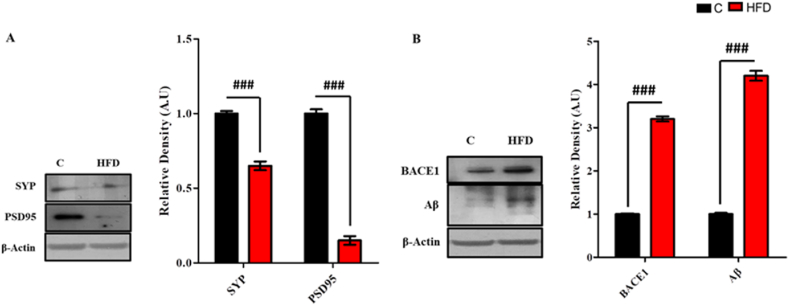
**HFD induced significant synaptotoxicity and amyloid burden in the hippocampus of the Dams.** Shown are the Western blot results of maternal (A) synapse receptor proteins (SYP and PSD-95) and (B) amyloidogenic burdon proteins (BACE-1 and Aβ) in pre-pregnancy stage, along with their bar-chart representation respectively. The assay was repeated three times. β-Actin was used as a loading control. The results were determined using Image J software and bar-chart indicate mean in A.U ± SEM. Significance of control vs HFD is expressed as #. Significance: ##p < 0.01 and ###p < 0.001.

Maternal obesity/HFD induced an obvious decrease in the synaptic receptor proteins and increased amyloidogenic burden. Significant decrease (p < 0.001) in the expression of synaptic proteins (SYP and PSD95) (Fig. 2A), and significant increase (p < 0.001) was observed in the expression of BACE-1 and Aβ in HFD group as compared to normal diet group (Fig. 2B).

After feeding the female mice with HFD for three weeks, the animals showed a significant increase in body weight accompanied by, deranged lipid profiles, oxidative stress and altered behavior as shown in the Supplementary Figs. S–3.

### 3.2 Melatonin exhibits reversal effect on HFD induced complications in the dams after parturition and post weaning interventions

**Fig. 3:**
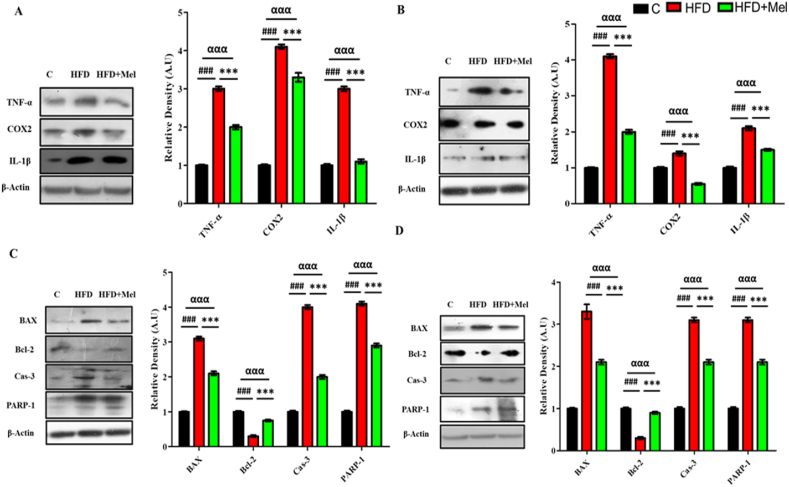
**Melatonin showed significant improvement in the expression levels of neuro-inflammatory and neuro-degeneration markers in HFD fed Dams.** Shown are the Western blot results of neuro-inflammatory markers (COX2, TNF-α and IL-1β) in (A) post parturition, and (B) post weaning stage, and neurodegeneration markers (BAX, Bcl-2, caspase-3 and PARP-1) in (C) post parturition, and (D) post weaning stage, along with their bar-chart representation respectively. The assay was repeated three times. β-Actin was used as a loading control. The results were determined using Image J software and bar-chart indicate mean in A.U ± SEM. Significance of one way ANOVA is expressed as α, significance of control vs HFD is expressed as #, while significance of HFD vs HFD + Mel is expressed as*. Significance: ^αα,^**^, ##^p < 0.01 and ^ααα,^***^, ###^p < 0.001.

We evaluated the neuroprotective potential of melatonin against HFD induced OS and associated neuroinflammation and neurodegeneration in the hippocampus of the mice brains. Statistically significant differences (p < 0.001) were observed in the expression of neuroinflammatory and neurodegenerative markers. Post hoc comparison revealed that HFD induced significant increase (p < 0.001) in the expression of neuroinflammatory markers (TNF-α, IL-1β and COX2). However, melatonin significantly decreased (p < 0.001) in all the proinflammatory markers (TNF-α, IL-1β and COX2) in both post-parturition (Fig. 3A) and post-weaning intervention (Fig. 3B). Similarly, HFD markedly increased BAX, Cas-3 and PARP-1 (p < 0.001) and decreased Bcl-2(p < 0.001), while melatonin significantly reversed (p < 0.001) BAX, Bcl-2, Cas-3 and PARP-1 expression in post-parturition (Fig. 3C) and post-weaning interventions (Fig. 3D).

**Fig. 4:**
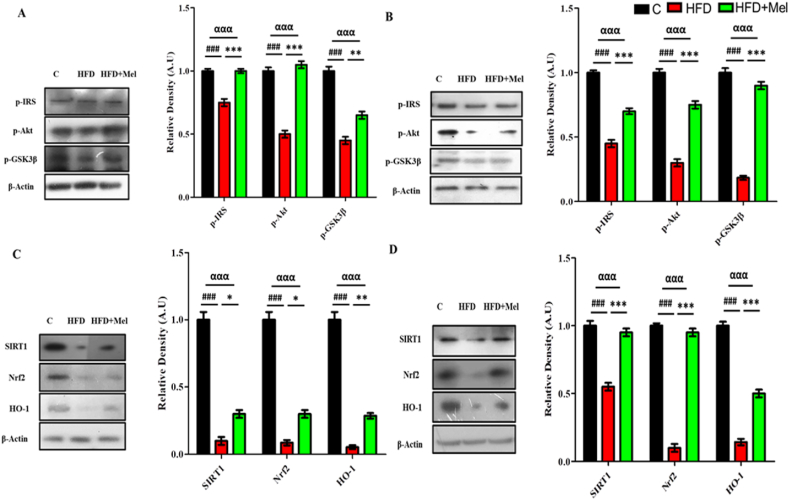
Melatonin demonstrated substantial amelioration in the central insulin resistance signaling pathway proteins expression. Shown are the Western blot results of insulin resistance markers (p-IRS, p-Akt and p-GSK3β) in (A) post parturition, and (B) post weaning stage, and signaling proteins (SIRT1, Nrf-2 and HO-1) in (C) post parturition, and (D) post weaning stage, along with their bar-chart representation respectively. The assay was repeated three times. β-Actin was used as a loading control. The results were determined using Image J software and bar-chart indicate mean in A.U ± SEM. Significance of one way ANOVA is expressed as α, significance of control vs HFD is expressed as #, while significance of HFD vs HFD + Mel is expressed as*. Significance: ^αα,^**^, ##^p < 0.01 and ^ααα,^***^, ###^p < 0.001.

Significant differences (p < 0.001) were also observed in the expression of insulin resistance markers (IRS-1, p-Akt and p-GSK3β). Post hoc tukey's analysis showed significant decrease (p < 0.001) in the expression of IRS-1, p-Akt and p-GSK3β proteins in the hippocampal homogenates of HFD group as compared to control group in both maternal interventions. Melatonin, however showed significant increase (p < 0.001) in the expression of almost all the assessed markers (IRS-1, p-Akt and p-GSK3β) in post-parturition intervention (Fig. 4A) in both maternal interventions (Fig. 4A and B).

Melatonin activated the SIRT1/Nrf2 pathway to reduce HFD induced OS. Nrf-2 and HO-1 along with SIRT-1 showed a significant decrement (p < 0.001) in the HFD group compared to control group. Melatonin, on the other hand markedly increased (p < 0.05) the expression of the signaling pathway proteins (SIRT1/Nrf2/HO-1) in the post-parturition intervention (Fig. 4C). However, highly significant increase (p < 0.001) was observed in the expression levels of SIRT1, Nrf2 and HO-1 in the post-weaning intervention (Fig. 4D).

**Fig. 5:**
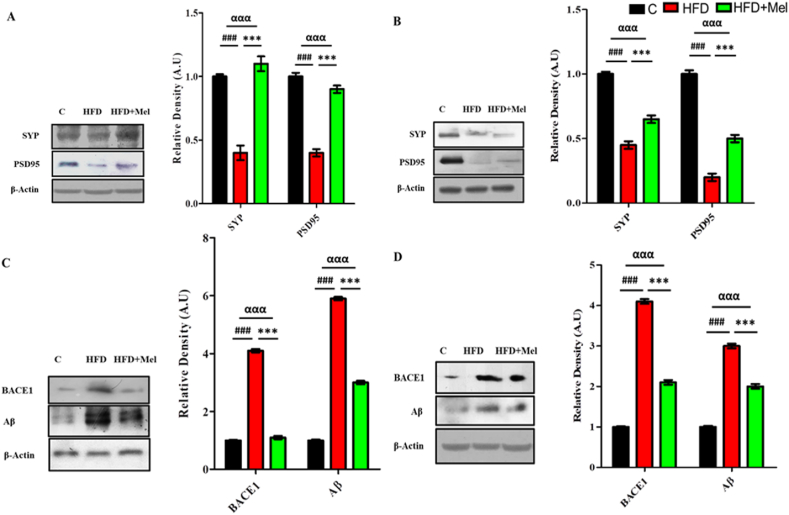
**Melatonin exhibited significant retrieval in the HFD-induced synaptotoxicity and amyloid burden in the dams' brain** Shown are the Western blot results of synapse receptor proteins (SYP and PSD-95) in (A) post parturition, and (B) post weaning stage, and amyloidogenic burdon proteins (BACE-1 and Aβ) in (C) post parturition, and (D) post weaning stage, along with their bar-chart representation respectively. The assay was repeated three times. β-Actin was used as a loading control. The results were determined using Image J software and bar-chart indicate mean in A.U ± SEM. Significance of one way ANOVA is expressed as α, significance of control vs HFD is expressed as #, while significance of HFD vs HFD + Mel is expressed as*. Significance: ^αα,^**^, ##^p < 0.01 and ^ααα,^***^, ###^p < 0.001.

HFD significantly decreased (p < 0.001) synaptic receptor proteins (SYP & PSD95) and significantly increased amyloid burden proteins (BACE-1 and Aβ) in both interventions. Melatonin, however markedly increased the expression of SYP (p < 0.01) & PSD95 (p < 0.001) (Fig. 5A and B) and decreased (p < 0.001) BACE-1 and Aβ in the mice brains in both post-parturition and post-weaning interventions (Fig. 5C and D).

**Fig. 6:**
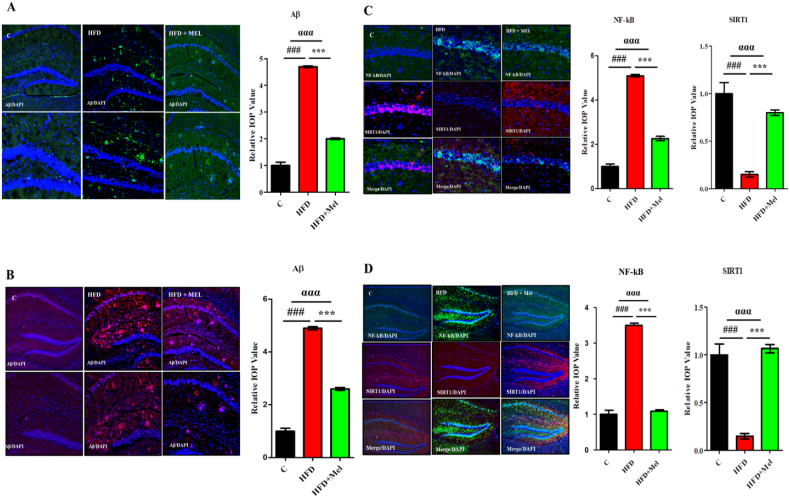
Immunohistochemistry analysis of melatonin showing immense amelioration of selected proteins in post-parturition and post-weaning stage of the dams. Shown are the (A) The immunohistochemistry images of Aβ (green) along with the relative integral optical density (IOD) bar chart, in the CA1 region of the hippocampus in experimental groups in post parturition stage, (B) the immunohistochemistry images (pink) of Aβ along with the relative integral optical density (IOD) bar chart, in the CA1 region of the hippocampus in the experimental groups in post-weaning stage. Also shown are the (C) the double immunohistochemistry images of NF-kB (green) and SIRT1 (red) along with the relative integral optical density (IOD) bar chart, in the CA1 region of the hippocampus in experimental groups in post parturition stage, and (D) the double immunohistochemistry images of NF-kB (green) and SIRT1 (red) along with the relative integral optical density (IOD) bar chart, in the CA1 region of the hippocampus in experimental groups in post weaning stage. DAPI was used to stain the nucleus. The complete procedures are depicted in the material and methods section. Significance of one way ANOVA is expressed as α, significance of control vs HFD is expressed as #, while significance of HFD vs HFD + Mel is expressed as*. Significance: ^ααα^P < 0.001, ^###^P < 0.001, ***P < 0.001.

Selected proteins Aβ, NF-kB and SIRT1 expressions were analyzed through immunohistochemistry technique. Significant differences (p < 0.001) were revealed in the expression of these proteins among various groups. Post hoc tukey test showed significant increase (p < 0.001) in the expression of Aβ protein in both post-parturition and post-weaning maternal interventions (Fig. 6A and B). Similarly, significant increase (p < 0.001) in the expression of NF-κB and significant decrease (p < 0.001) in SIRT1 expression was observed in HFD group as compared to normal chow diet group in both maternal interventions (Fig. 6C and D). Melatonin treatment significantly rectified (p < 0.001) the effect of HFD in both post-parturition and post-weaning interventions.

### 3.3 Maternal melatonin treatment protects mice offspring from the deteriorating effects of HFD

**Fig. 7:**
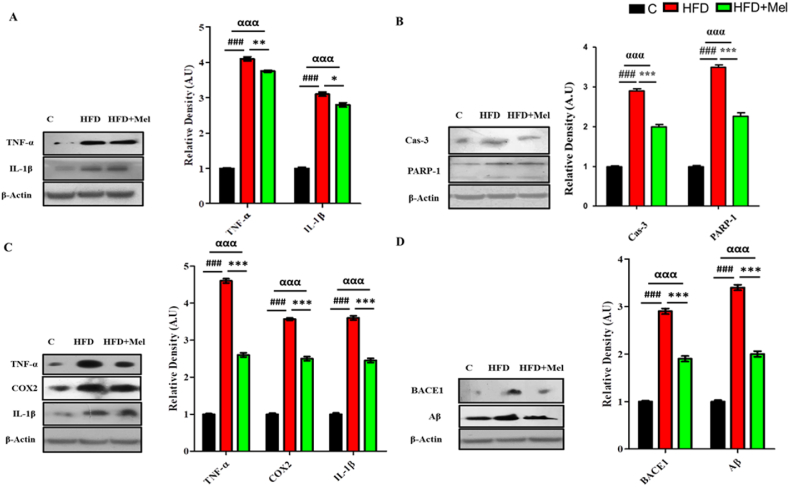
**Melatonin successfully ameliorated the expression levels of various proteins in the young and juvenile offspring brains of the HFD group.** Shown are the Western blot results of (A) neuroinflammatory markers (TNF-α and IL-1β) and (B) neurodegeneration markers (Cas-3 and PARP-1) along with their bar chart respectively in the neonatal brains of the HFD group. Also shown are the Western blot results of (C) neuroinflammatory markers (COX2 and TNF-α) and (D) amyloid burdon (BACE-1 and Aβ) along with their bar chart respectively in the HFD group juvenile offspring. The assay was repeated three times. β-Actin was used as a loading control. The results were determined using Image J software and bar chart indicate mean in A.U ± SEM. Significance of one way ANOVA is expressed as ^α^, significance of control vs HFD is expressed as #, while significance of HFD vs HFD + Mel is expressed as*. Significance: ^αα,^**^, ##^p < 0.01 and ^ααα,^***^, ###^p < 0.001.

**Fig. 8:**
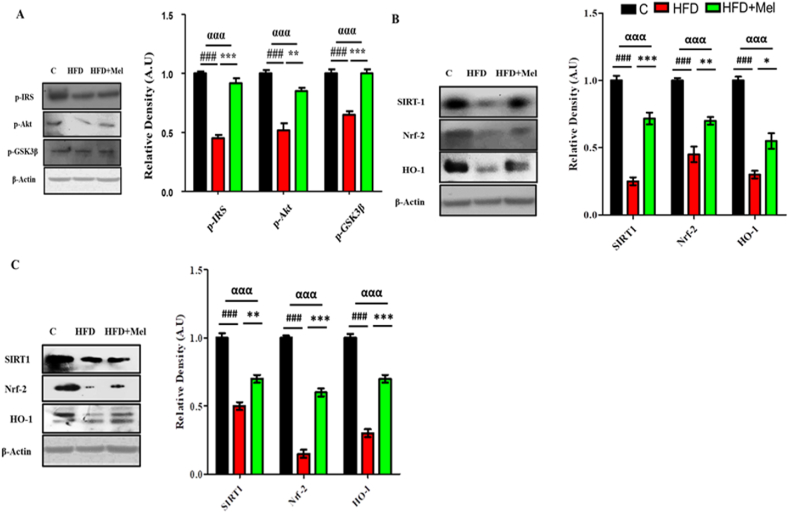
**Melatonin successfully rectified insulin resistance and enhance signaling proteins in in the young and juvenile offspring brains.** Shown are the Western blot results of (A) insulin resistance makers (p-IRS, p-Akt and p-GSK3β) and (B) signaling proteins (SIRT1, Nrf-2 and HO-1) along with their bar chart respectively in juvenile offspring. Also Shown are the Western blot results of signaling proteins (SIRT1, Nrf-2 and HO-1) along with their bar chart in the young offspring brains of the HFD group. The assay was repeated three times. β-Actin was used as a loading control. The results were determined using Image J software and bar chart indicate mean in A.U ± SEM. Significance of one way ANOVA is expressed as ^α^, significance of control vs HFD is expressed as #, while significance of HFD vs HFD + Mel is expressed as*. Significance: ^α,^*^,#^p < 0.05, ^αα,^**^, ##^p < 0.01 and ^ααα,^***^, ###^p < 0.001.

Maternal HFD and HFD + melatonin effects were observed in two offspring's interventions at the age of 4 and 8 weeks respectively. Post-hoc analysis showed significant increase (p < 0.001) in the expression of neuro-inflammatory markers (TNF-α and IL-1β) in the HFD groups of young offspring as compared to control group (Fig. 7A). Similarly, a significant increase (p < 0.001) was observed in the expression of neurodegeneratory markers (Cas-3 and PARP-1) in HFD group of juvenile offspring. Melatonin meticulously reversed all the damages caused by HFD. A significant decrease of pro-inflammatory markers, TNF-α (p < 0.01) and IL-1β (p < 0.05) was observed (Fig. 8A) along with a highly significant decrease (p < 0.001) in the neurodegeneratory markers in the brain homogenates of young offspring of HFD + Melatonin group (Fig. 7B).

Neuro-inflammatory and amyloidogenic markers were also investigated in the hippocampal homogenates of the juvenile offspring. Significant increase (p < 0.001) in the neuro-inflammatory markers i.e.; TNF-α, IL-1β and COX2 levels were observed in the HFD groups of juvenile offspring as compared to control group. Similarly, a significant increase (p < 0.001) was observed in the expression of BACE-1 and Aβ proteins in HFD group juvenile offspring as compared to control group. Melatonin successfully reversed all the damages caused by HFD. Post hoc tukey test showed highly significant decrease (p < 0.001) in the pro-inflammatory markers (TNF-α, IL-1β and COX-2) levels (Fig. 7C) along with significant decrease (p < 0.001) in the expression of BACE-1 and Aβ proteins in the brain homogenates of the HFD + melatonin group of juvenile offsprings (Fig. 7D).

Significant differences (p < 0.001) were observed in the expression of insulin resistance markers (p-IRS-1, p-Akt and p-GSK3β) among various groups of juvenile offspring. Significant decrease (p < 0.001) in the expression of p-IRS-1, p-Akt and p-GSK3β was observed in the HFD group juvenile offspring brain. Melatonin on the other hand significantly retrieved (p < 0.001) these markers (Fig. 8A).

SIRT-1 expression and activity was analyzed in both young and juvenile offspring. Significant differences (p < 0.001) were observed among the expression levels of SIRT-1 signaling proteins. Significant decrease (p < 0.001) in the expression of Nrf2 and HO-1 along with SIRT-1 levels in HFD group of both young and juvenile offspring were observed as compared to the control group. Melatonin however, successfully rectified this decrease in both the offspring interventions. Significant increase (p < 0.001) in SIRT-1, Nrf2 and HO-1 expression levels were observed in HFD + melatonin group of juvenile offspring (Fig. 8B). Similarly, melatonin significantly increased (p < 0.001) the expression levels of the signaling protein in HFD + melatonin group young offspring (Fig. 8C).

**Fig. 9:**
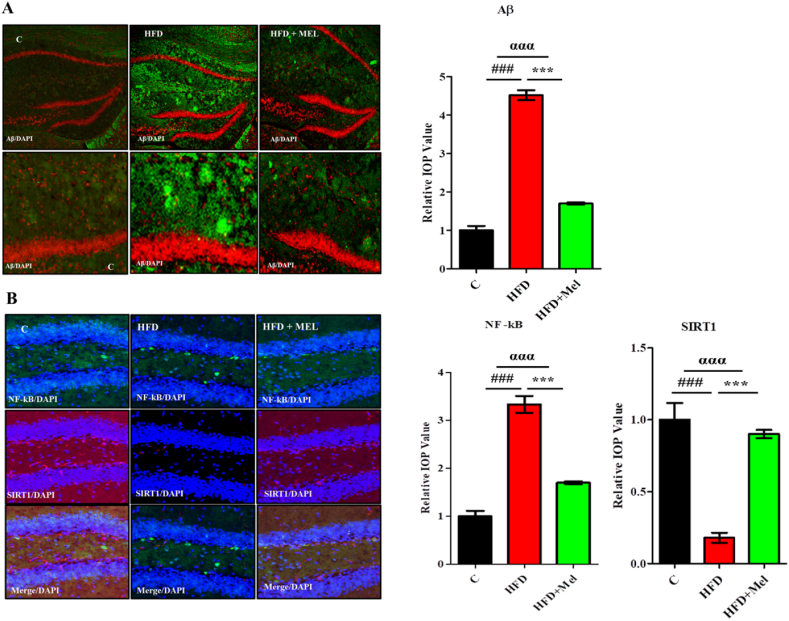
Immunohistochemistry analysis of melatonin showing immense amelioration of selected proteins in the CA1 region of hippocampus of the juvenile offspring. Shown are the (A) The immunohistochemistry images of Aβ (green) along with the relative integral optical density (IOD) bar chart, in the CA1 region of the hippocampus in juvenile offspring and (B) the double immunohistochemistry images of NF-kB (green) and SIRT1 (red) along with the relative integral optical density (IOD) bar charts, in the CA1 region of the hippocampus in juvenile offspring. DAPI was used to stain the nucleus. The complete procedures are depicted in the material and methods section. Significance of one way ANOVA is expressed as α, significance of control vs HFD is expressed as #, while significance of HFD vs HFD + Mel is expressed as*. Significance: αα, **, ##p < 0.01 and ααα, ***, ###p < 0.001.

Selected proteins NF-κB, SIRT1 and Aβ proteins expressions were analyzed through immunohistochemistry technique. Post hoc comparison demonstrate significant increase (p < 0.001) in the expression of the NF-kB and Aβ proteins and significant decrease (p < 0.001) in SIRT1 expression in HFD group. Melatonin treatment significantly improved (p < 0.001) the detrimental effects of HFD in the HFD + Melatonin group (Fig. 9A and B).

## 4 Discussion

The present study contributes to identify therapeutic effects of melatonin to treat HFD induced neurodegenerative diseases. HFD induced metabolic dysregulation in pregnancy plays a crucial role in the progression of neurological disorders not only in the female but it also increases the odds of metabolic misadventures in offspring. This study examined potential neuroprotective effects of melatonin against the detrimental effects of HFD on the brains of the dams and their upcoming generation. This mechanistic investigation provides the first evidence that melatonin alleviates oxidative stress and neuroinflammation induced by a HFD through an SIRT1-/Nrf2-dependent pathway in the brains of dams and their offspring.

In this study, chronic HFD feeding induced significant increase in the mice weight and caused metabolic dysregulation. Amin et al. (2011) indicated a significant increase in serum lipid profile (TC, TG and LDL levels) in HFD [28]. However, it is recently reported that four weeks melatonin treatment caused a significant decrease in body mass, cholesterol, LDL and triglycerides levels in HFD-induced obese mice [29]. Lowering of serum cholesterol and triglycerides by melatonin have been shown due to its antioxidant potential leading to reduced OS [30]. These results are consistent with our study showing the protective effect of melatonin on serum LDL, cholesterol and triglycerides levels against HFD in both maternal and offspring intervention.

In our study we analyzed HFD induced OS through commonly used oxidative stress markers like superoxide dismutase (SOD), peroxidase (POD), catalase (CAT), glutathione peroxidase (GSH), and lipid peroxidase (LPO) (detailed in the supplementary materials). Previous studies have shown that HFD produces an imbalance between ROS and antioxidant enzyme/species [31]. Overproduction of free radicals due to HFD consumption leads to cellular damage [32]. Alzoubi et al. (2018) reported that HFD increases OS in the hippocampal region by reducing GSH/GSSG ratio [31]. Markedly increased levels of TBARS have been observed in the cortex and hippocampus of the diabetic mice brain, fed on HFD, causing apoptosis of neuronal cells, along with learning and memory impairment [33]. In addition, it is previously shown that HFD causes impaired hippocampal neurogenesis via elevated lipid peroxidation [34]. In the present study, we evaluated different antioxidant enzyme assays in the homogenates of hippocampus and cortex regions of the experimental female mice exposed to normal chow and HFD respectively. The OS markers such as SOD, POD, CAT, and GSH were inhibited, while LPO was elevated in HFD fed groups as compared to the mice on normal standard diet. Treatment of HFD fed mice with melatonin ameliorates the damage caused by HFD during pregnancy. Melatonin was found effective in the reduction of OS by upregulating antioxidant enzymes such as POD, SOD, CAT, GSH, and reduced LPO activities in the hippocampus and cortex regions of the mothers and their offspring.

Neuroinflammation is initially a defensive response in the brain; however, excessive inflammatory reactions can be harmful and impede neuronal regeneration [35]. Interleukin-1 (IL-1) is released in response to cellular damage, infections, and inflammatory conditions by monocytes and macrophages along with non-immune cells like fibroblasts and endothelial cells [36]. Tumor necrosis factor-alpha (TNF-α) influences various signaling pathways to regulate apoptosis, whereas Cyclooxygenase (COX) also known as prostaglandin synthase, is a potent mediator in inflammation [37]. Chronic HFD consumption subsequently induces inflammation. During the progression of obesity, adipocytes play a role in promoting inflammation by releasing typical proinflammatory cytokines, including IL-1, IL-6, COX-2, and TNF-α. These cytokines are capable of crossing the blood-brain barrier, triggering inflammation in response to diverse factors [38].

Previous studies have shown that during acute neuroinflammation, NF-κB moves to the nucleus, attaches to DNA, and triggers the expression of proinflammatory markers like IL-1, IL-6, TNF-α, and COX-2 [38,39]. Furthermore, HFD-induced ROS introduces the activation and translocation of NF-κB by mobilizing proinflammatory cytokines [[40], [41]]. Also the up-regulation of TNF-α levels in the brains of mice fed with HFD, confirms neuroinflammation in the diet-induced obesity in animal model [42]. Concomitant with the previous studies, present study also shows that HFD promotes neuroinflammation in the hippocampus, as indicated by increased TNF-α, IL-1β and COX2 levels in this region.

The advent and progression of neurodegenerative diseases are linked to chronic neuroinflammation. Different studies have revealed the relationship between neurodegenerative diseases and hippocampal neuronal loss due to apoptosis [43]. The active (cleaved) form of caspase-3 is translocated to the nucleus, where it cleaves poly (ADP-ribose) polymerase (an enzyme involved in DNA repair), and thus leads to apoptosis [44]. This further leads to mitochondrial translocation of the pro-apoptotic protein Bax and subsequent release of cytochrome *c* from intermembrane space followed by caspase-3 activation, whereas Bcl-2 protein inhibits apoptosis [44]. Numerous studies have demonstrated that melatonin influences proinflammatory cytokines and has anti-inflammatory effects in various pathological conditions [[45], [46]]. Liu et al. (2018) found that melatonin diminishes LPS-induced inflammation in mouse adipose tissue by regulating inflammasome gene expression [47]. In our study, melatonin significantly lowered levels of TNF-α, IL-1β, and COX-2, as well as cleaved caspase-3, PARP-1, and Bax proteins, while increasing Bcl-2 protein expression in both maternal and offspring interventions.

The consumption of HFD, along with chronic stress, sleep deprivation, and aging, significantly exacerbates cognitive decline. Various studies have highlighted that diets rich in refined sugar and saturated fats detrimentally impact cognitive functions [47,48]. Post-synaptic density protein (PSD-95) is a scaffolding protein, which functions as an N-methyl-D-aspartate (NMDA) glutamate receptor-associated protein in central synapses. This NMDA receptor complex plays a pivotal role in synaptic plasticity and memory formation [[49], [50]]. Synaptophysin (SYP), a calcium-binding glycoprotein found in presynaptic vesicle membranes, is crucial for vesicular trafficking, synaptogenesis, docking, synaptic reorganization, and vesicular fusion with the synaptic plasma membrane. Decreased expression of PSD-95 and synaptophysin proteins are linked to memory impairment [51]. Amyloid plaques, composed of β-amyloid (Aβ) peptides derived from the amyloid precursor protein (APP) through sequential cleavage by β- and γ-secretase, play a significant role in neurodegeneration. The enzyme BACE-1, a β-secretase, cleaves APP at the extracellular membrane domain, producing soluble APP-β [52]. Uranga et al. (2010) reported that HFDs can exacerbate age-related cognitive decline [53]. Similarly, HFD consumption for one week [54] and 21 weeks impairs recognition memory [55]. We also observed HFD induced OS mediated synaptic dysfunction and increased amyloidogenic burden in all the maternal and adult intervention along with gross decline in the expression of synaptic proteins SYP and PSD95 and significant increase in BACE-1 and Aβ expression. Mihardja et al. (2020) observed that melatonin administration in APP695 transgenic mice reduced brain Aβ levels [56]. Although the exact underlying mechanism is unclear, it is proposed that melatonin may directly affect the metabolism of β-amyloid precursor protein. In our study, administration of melatonin during pregnancy has long lasting effect on restoring memory, synaptotoxicity and amyloid burden by significantly increasing, synaptic proteins (SYP and PSD95) and decreasing amyloid protein (BACE1 and Aβ) in the brain homogenates of hippocampus.

In the current study, we also assessed the detrimental effect of HFD on the cognitive abilities of pregnant mice and their offspring along with the ameliorative effects of melatonin through various behavioural tests. Xiong et al. (2022) demonstrated that HFD induced obesity is associated with cognitive impairment in mice [57]. In MWM tests, chronic HFD fed pR5 mice had increased escape latency time, indicating impaired learning ability [57]. Probe test showed pR5 mice on HFD spent less time in the designated platform quadrant, indicating impairment in spatial reference memory as compared to controls [57]. In our study, both short and long term memory impairments were observed by Y-maze and MWM tests, high escape latency time and decrease time duration, respectively in HFD fed mice and adolescents as compared to normal chow fed animal groups. Similarly, the percentage of spontaneous alternation in the Y-maze test was less in HFD fed female mice. According to Ahmad et al. (2014), decrease in spatial reference memory in mice may be due to aggregation of tau in the hippocampus and amygdala [58]. Melatonin showed improvement in memory in both MWM and Y-maze tests respectively in mothers and adolescent mice.

Multiple markers like phosphorylated insulin receptor substrate 1(p-IRS), protein kinase B (p-Akt) and phosphorylated glycogen synthase kinase-3 beta (p-GSK3β) are quantified for the insulin resistance and diabetes mellitus in this study. Previous studies have established a strong link between high saturated fat diets and the prevalence of obesity, type 2 diabetes mellitus [59] and insulin resistance [60,61]. For instance, female C57BL/6J mice fed on different fat percentages diet over 15 weeks exhibited a linear correlation between dietary fat percentage and glucose intolerance [62]. McNeilly et al. (2015) also reported increased fasting blood glucose and insulin levels in rats fed on HFD (45 % of calories from fat) [63]. Similarly, Kothari et al. (2017) also found that HFD induced both peripheral and central insulin resistance [9]. Central insulin resistance was further linked to amyloid-beta (Aβ) deposition, neurofibrillary tangle formation, and reduced synaptic plasticity [64]. On the other hand, Owino et al. (2019) showed that melatonin activates MT2 receptors in human adipocytes, promoting glucose uptake [65]. Consistent with prior studies, our results revealed impaired insulin signaling and a significant reduction in these marker expressions. However, melatonin treatment effectively restored the levels of p-IRS, p-Akt, and p-GSK3β proteins in all maternal and offspring interventions.

The silent information regulator transcript-1 (SIRT1) involved in the regulation of numerous processes, like metabolism, apoptosis, deacetylating histones and nonhistone proteins and inflammation. Several studies have revealed that SIRT1 reduces OS level and the extent of inflammation [65,66] Nrf2 is a key antioxidant protein essential for cellular defense, when activated moves from the cytoplasm to the nucleus, it interacts with the antioxidant defense system to stimulate the transcription of hemeoxygenase 1 (HO-1) [67]. This activation enhances resistance to OS and provides protection against inflammation [68]. Nawaz et al. (2018) found that HFD deactivates SIRT1 in adipocytes, leading to various transcriptional changes [69] and melatonin activates the SIRT1/Nrf2 signaling pathway against LPS-induced OS in the hippocampus of 7-day postnatal rats [22]. In accordance with the previous studies, our study also revealed that HFD significantly inhibited the expression of SIRT1 and the endogenous antioxidant response via Nrf2 and HO-1 proteins, in the experimental mice brains. However, melatonin administration activated Nrf2/HO-1 signaling proteins, in an SIRT1-dependent manner to rescue mice’ brain against HFD induced OS and acute neuroinflammation in both maternal and offspring interventions.

One limitation of this study is that, we only observed the therapeutic effects of melatonin in the offspring groups of their own respective maternal groups. Secondly, cross-fostering of the neonates was not possible because mothers used to eat pups from the other groups despite having free access to food and water. Moreover, we were unable to check the inflammatory markers in peripheral tissues because of the financial constraints of the study.

## 5 Conclusion

We conclude that HFD induced neuro-inflammation and neuro-degeneration in pregnant mice and their offspring leads to oxidative stress. Oxidative stress further triggered insulin resistance and amyloid-β generation in the brains of pregnant mice and their offspring. Melatonin rectified the damaging effect caused by HFD via activating SIRT1/Nrf-2/HO-1 signaling pathway. Since limited remedies are available to effectively treat HFD induced brain damages, this study could be helpful in the development of potential treatment strategies in obese pregnant mothers and their offspring.

## Funding information

This research was supported by Organization of Research Innovation and commercialization of 10.13039/100031373Khyber Medical University and the publication charges for this article are partially borne from 10.13039/100031373Khyber Medical University publication fund (reference No. DIR/ORIC/REF/22/00003).

## Data availability

Available from the corresponding author on reasonable request.

## CRediT authorship contribution statement

**Amin Jan:** Writing – original draft, Visualization, Investigation, Formal analysis. **Mohsin Shah:** Writing – review & editing, Funding acquisition, Conceptualization. **Shahid Ali Shah:** Writing – review & editing, Investigation, Formal analysis. **Syed Hamid Habib:** Writing – review & editing. **Ehtesham Ehtesham:** Writing-review & editing. **Naseer Ahmed:** Writing – review & editing.

## Declaration of competing interest

The authors declare that they have no known competing financial interests or personal relationships that could have appeared to influence the work reported in this paper.
