# Supplementary material for: Melatonin rescues pregnant female mice and their juvenile offspring from high fat diet-induced alzheimer disease neuropathy
Source: Heliyon. 2024 Aug 24;10(17):e36921. doi: 10.1016/j.heliyon.2024.e36921 (PMC11395765; doi:10.1016/j.heliyon.2024.e36921)
Supplement: Multimedia component 1 [file mmc1.docx]

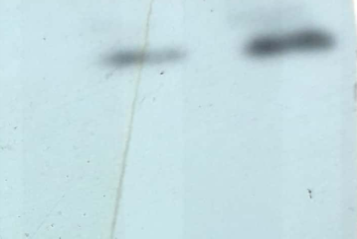

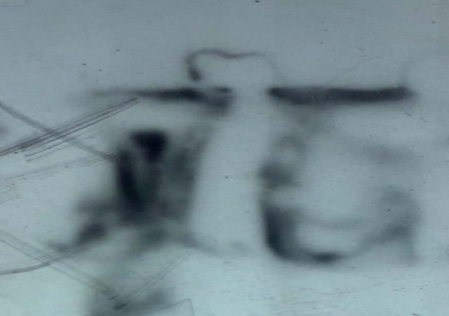

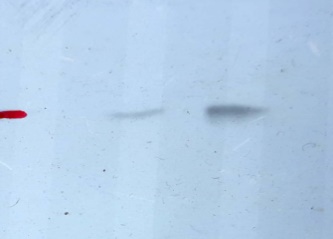


**1A**

**IL-1β**

**COX-2**

**TNF-α**

**Figure 1**

**PARP-1**

**Cas-3**

**Bcl-2**

**BAX**


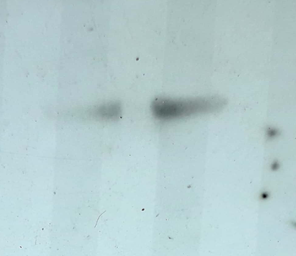

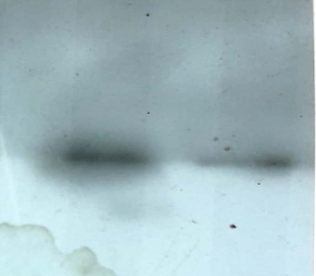

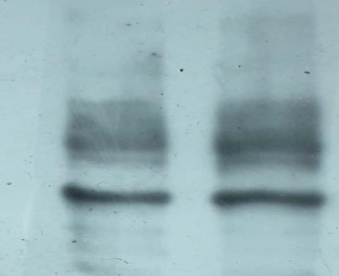

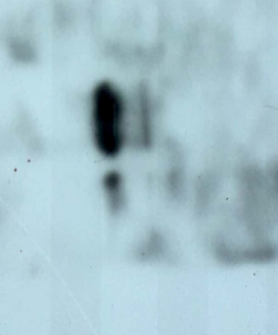


**1B**

**p-GSK3β**

**p-IRS**

**p-Akt**


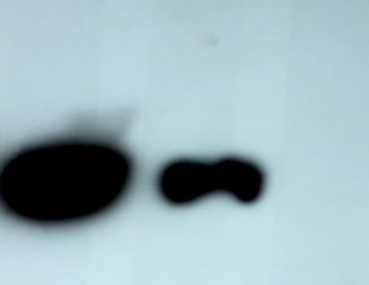

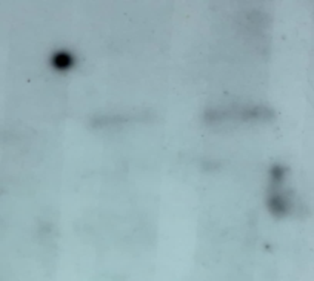

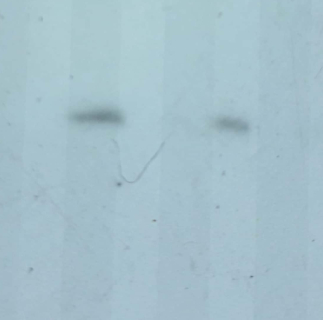


**1C**

**HO-1**

**Nrf2**

**SIRT-1**


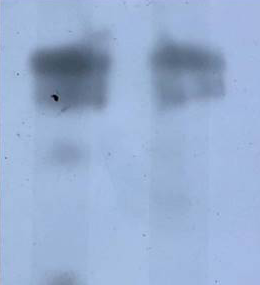

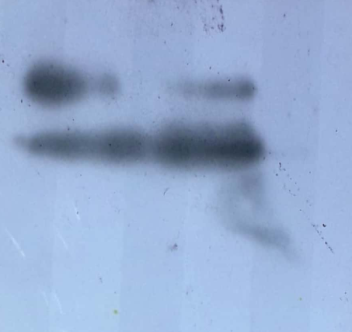

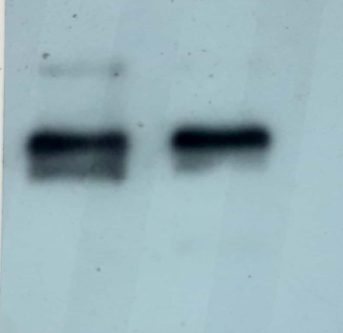


**1D**

**Figure 1: HFD induced significant changes in the expression levels of various markers in the hippocampus of the Dams.**

Shown are the Western blot results of maternal (A) neuroinflammatory markers (COX2, TNF-α and IL-1β), (B) neurodegeneration markers (BAX, Bcl-2, Cas-3 and PARP-1), (C) insulin resistance markers (p-IRS, p-Akt and p-GSK3β) and (D) signaling proteins (SIRT1, Nrf2 and HO-1) in pre-pregnancy stage.

**Figure 2**

**PSD-95**

**SYP**


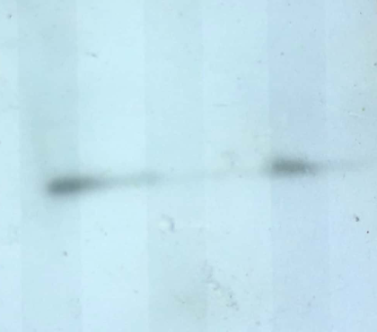

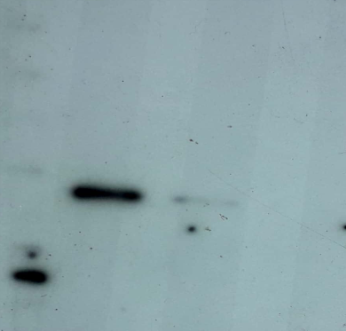


**2A**

**BACE-1**

**Aβ**


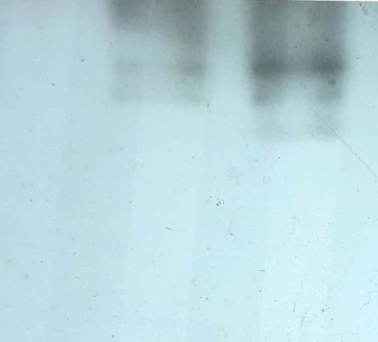

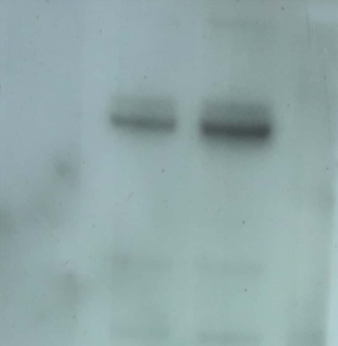


**2B**

**Figure 2: HFD induced significant** **synaptotoxicity and amyloid burden in the hippocampus of the Dams.**

Shown are the Western blot results of maternal (A) synapse receptor proteins (SYP and PSD-95) and (B) amyloidogenic burdon proteins (Aβ and BACE-1) in pre-pregnancy stage.

**Figure 3**

**COX-2**

**IL-1β**

**TNF-α**


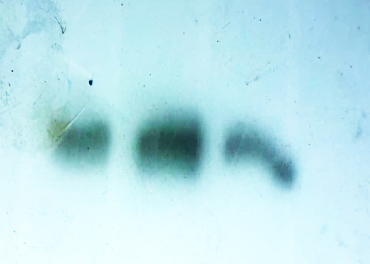

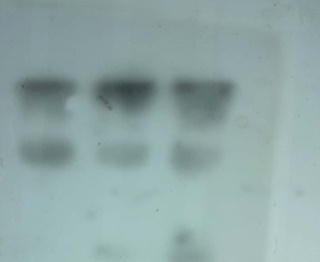

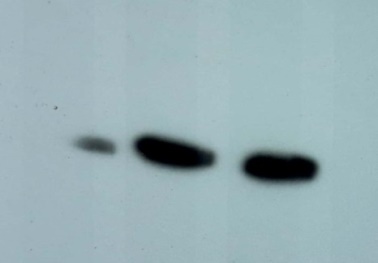


**3A**

**TNF-α**

**IL-1β**

**COX-2**


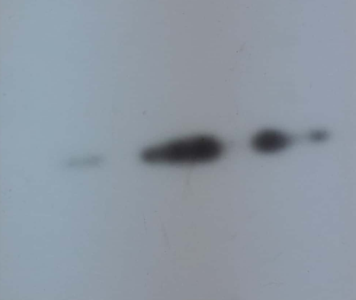

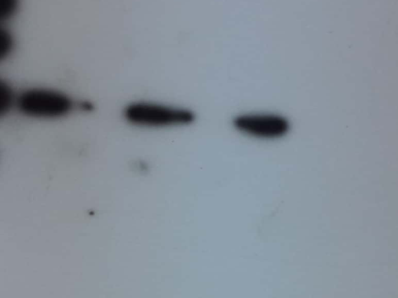

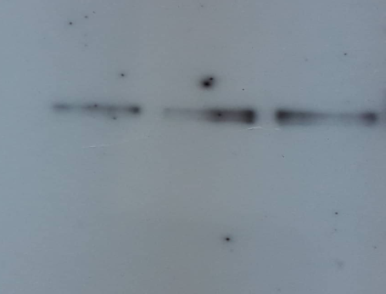


**3B**

**Bcl-2**

**Cas-3**

**PARP-1**

**BAX**


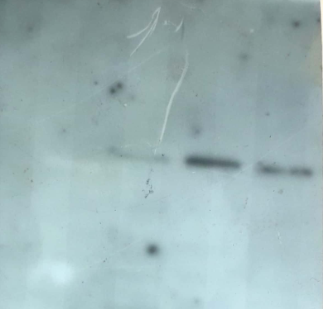

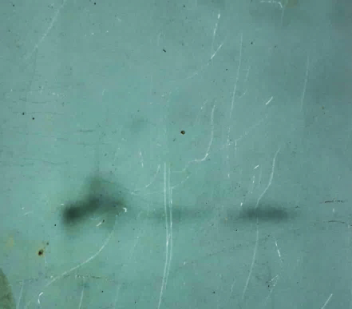

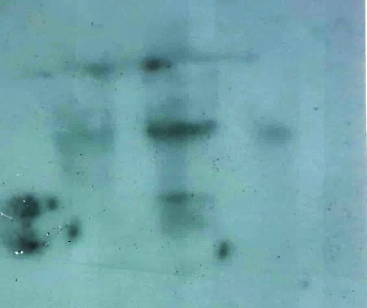

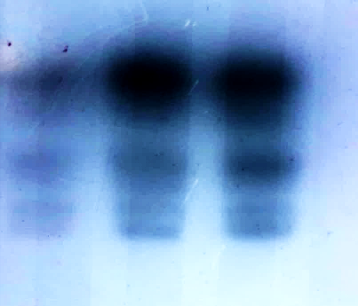


**3C**

**PARP-1**

**Cas-3**

**Bcl-2**

**BAX**


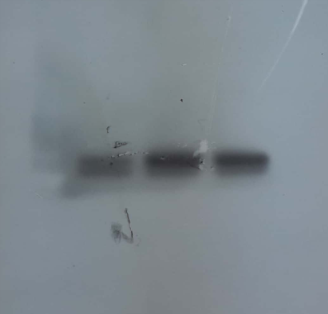

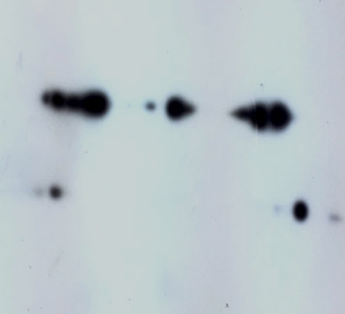

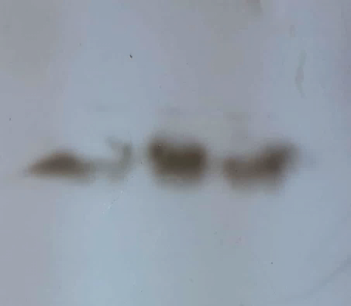

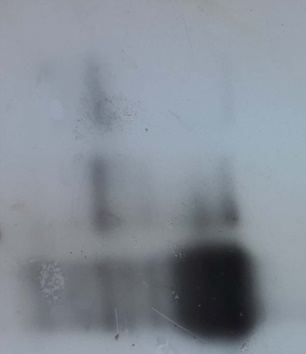


**3D**

**Figure 3:** **Melatonin showed significant improvement in the expression levels of neuro-inflammatory and neuro-degeneration markers in HFD fed Dams.**

Shown are the Western blot results of neuro-inflammatory markers (COX2, TNF-α and IL-1β) in (A) post parturition, and (B) post weaning stage, and neurodegeneration markers (BAX, Bcl-2, caspase-3 and PARP-1) in (C) post parturition, and (D) post weaning stage.

**Figure 4**

**p-GSK3β**

**p-Akt**

**p-IRS**


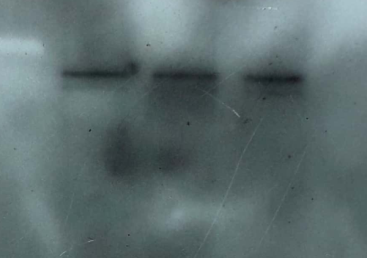

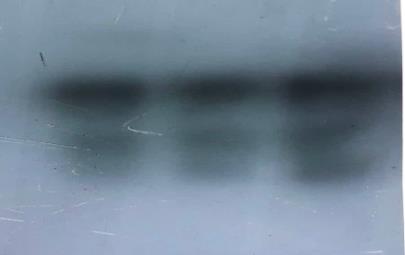

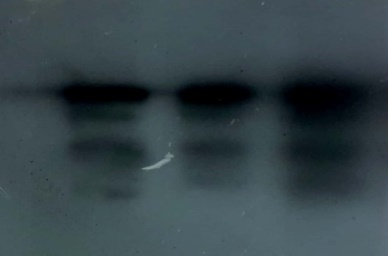


**4A**

**p-GSK3β**

**p-Akt**

**p-IRS**


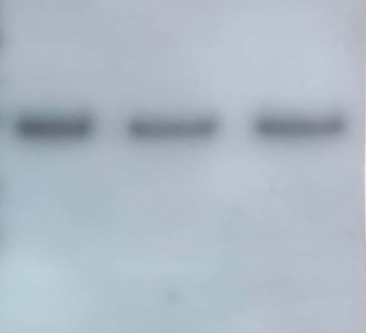

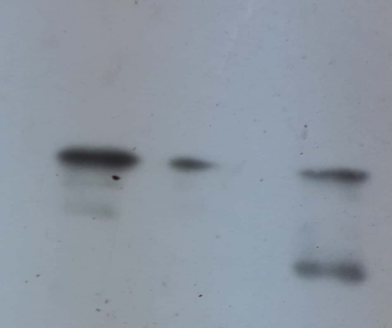

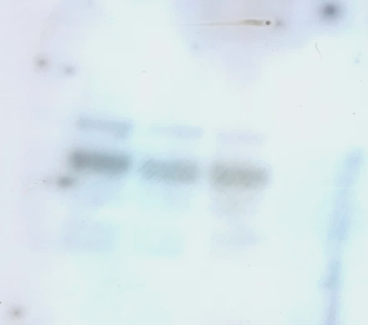


**4B**

**HO-1**

**Nrf2**

**SIRT1**


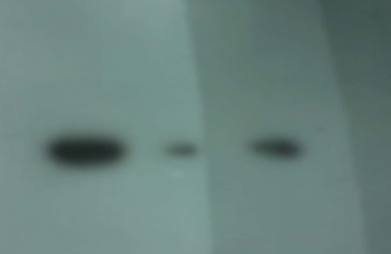

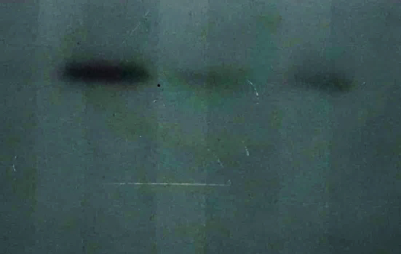

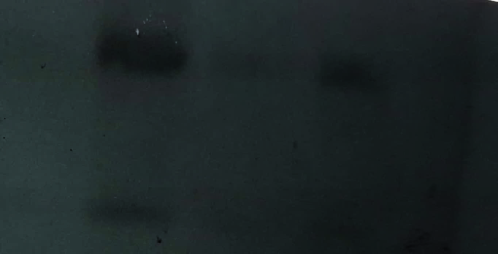


**4C**

**HO-1**

**Nrf2**

**SIRT1**


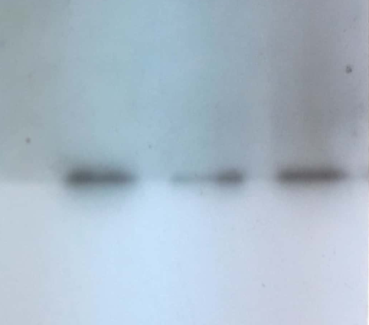

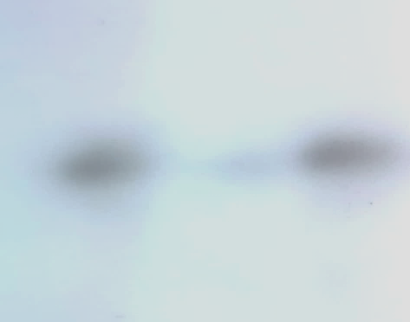

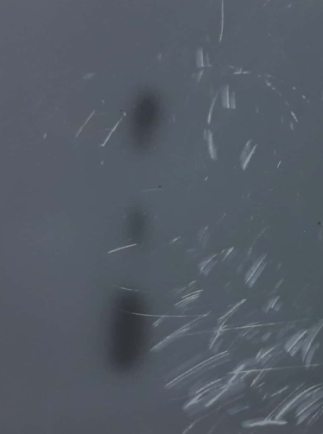


**4D**

**Figure 4: Melatonin demonstrated substantial amelioration in the central insulin resistance signaling pathway proteins expression.**

Shown are the Western blot results of insulin resistance markers (p-IRS, p-Akt and p-GSK3β) in (A) post parturition, and (B) post weaning stage, and signaling proteins (SIRT1, Nrf-2 and HO-1) in (C) post parturition, and (D) post weaning stage.

**Figure 5**

**PSD-95**

**SYP**


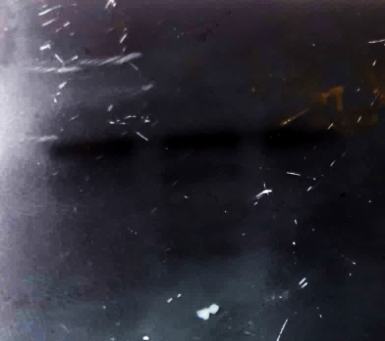

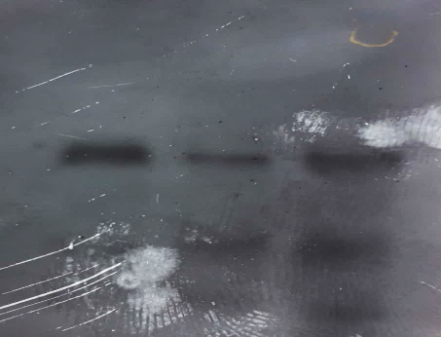


**5A**

**PSD-95**

**SYP**


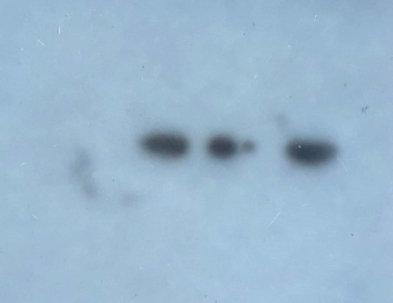

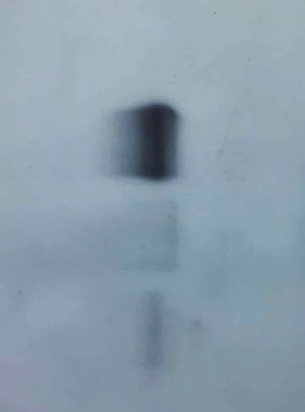


**5B**

**Aβ**

**BACE-1**


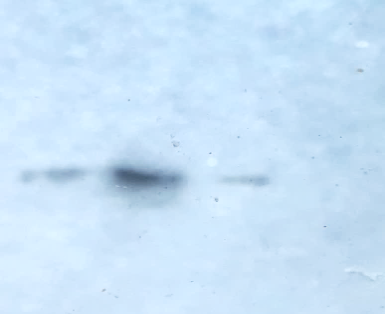

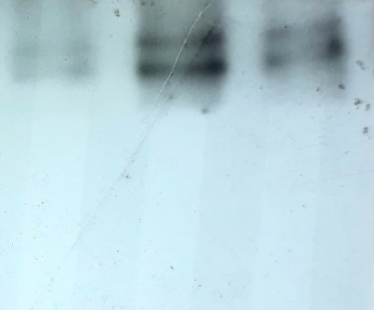


**5C**

**Aβ**

**BACE-1**


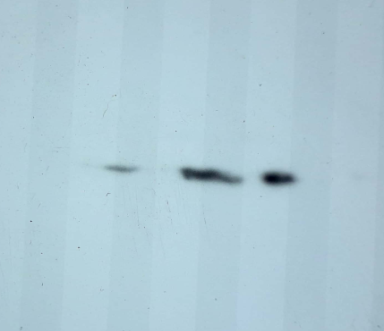

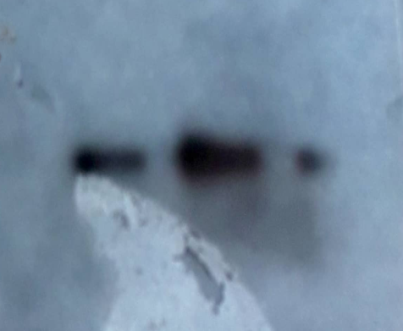


**5D**

**Figure 5:** **Melatonin exhibited significant retrieval in the HFD-induced synaptotoxicity and amyloid burden in the dams’ brain**

Shown are the Western blot results of synapse receptor proteins (SYP and PSD-95) in (A) post parturition, and (B) post weaning stage, and amyloidogenic burdon proteins (BACE-1 and Aβ) in (C) post parturition, and (D) post weaning stage.

**Figure 7**

**TNF-α**

**IL-1β**


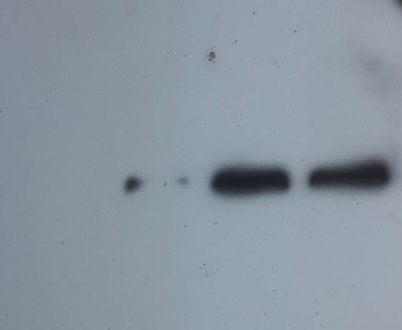

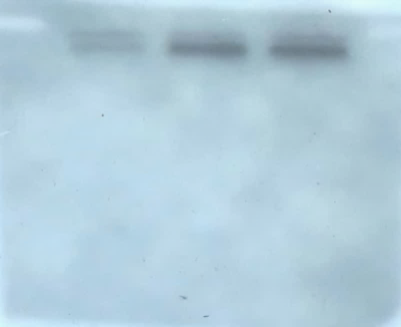


**7A**

**PARP-1**

**Cas-3**


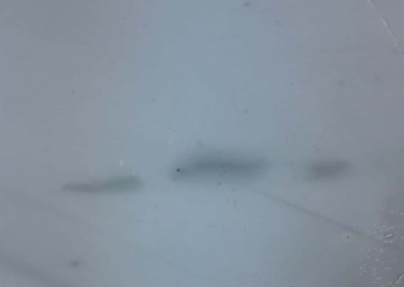

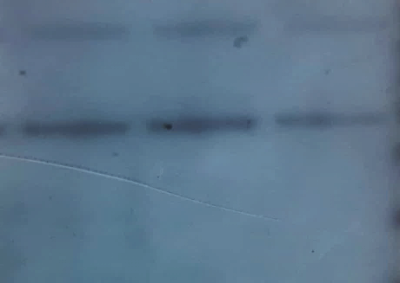


**7B**

**IL-1β**

**COX-2**

**TNF-α**


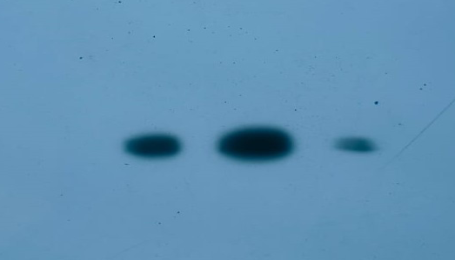

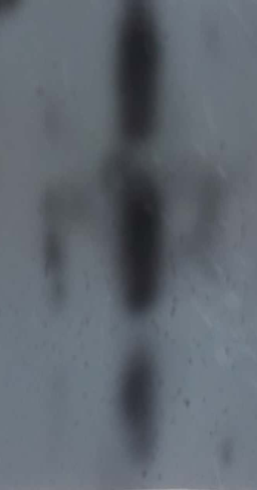

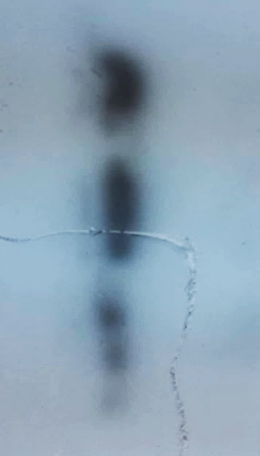


**7C**

**Aβ**

**BACE-1**


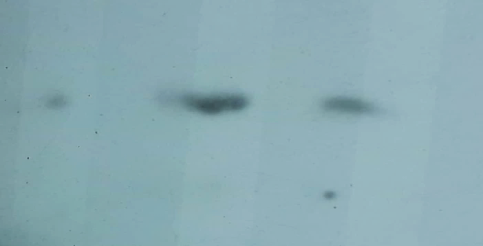

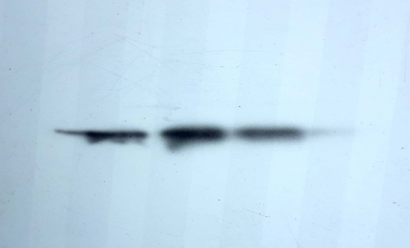


**7D**

**Figure 7:** **Melatonin successfully ameliorated the expression levels of various proteins in the young and juvenile offspring brains of the HFD group.**

Shown are the Western blot results of (A) neuroinflammatory markers (TNF-α and IL-1β) and (B) neurodegeneration markers (Cas-3 and PARP-1) in the neonatal brains of the HFD group. Also shown are the Western blot results of (C) neuroinflammatory markers (COX2 and TNF-α) and (D) amyloid burdon (BACE-1 and Aβ) in the HFD group juvenile offspring.

**Figure 8**

**p-GSK3β**

**p-IRS**

**p-Akt**


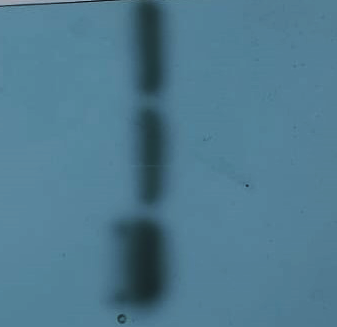

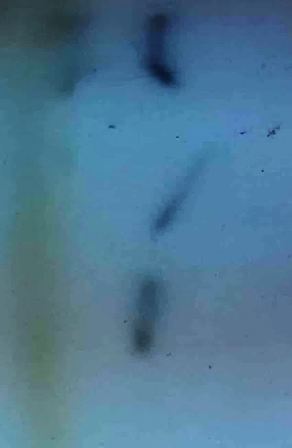

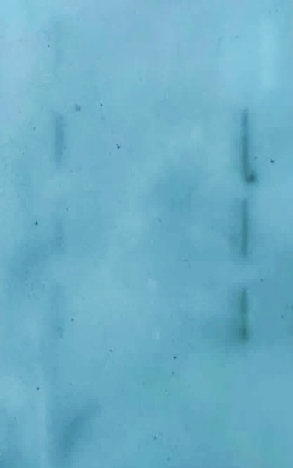


**8A**

**HO-1**

**Nrf2**

**SIRT1**


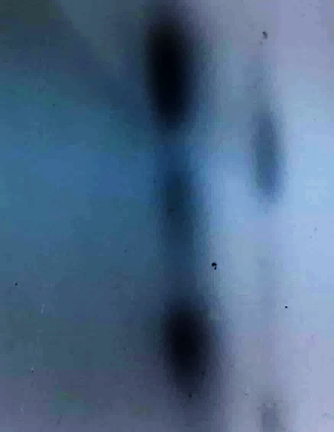

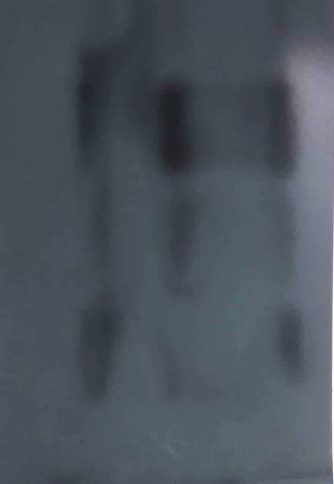

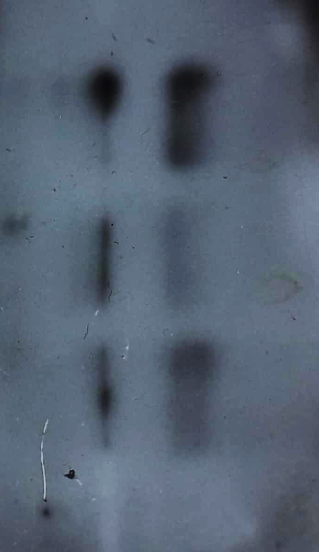


**8B**

**HO-1**

**Nrf2**

**SIRT1**


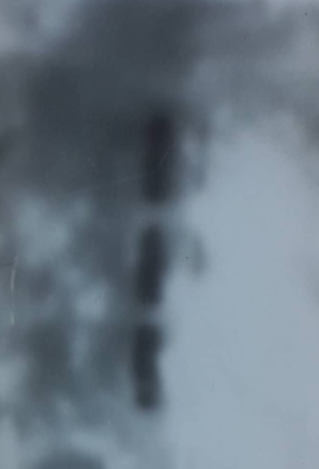

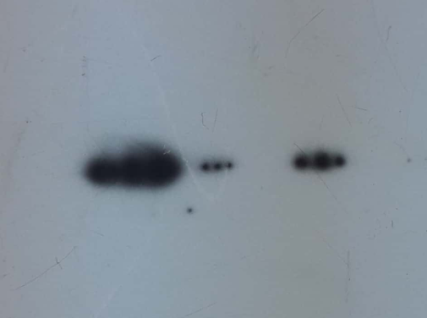

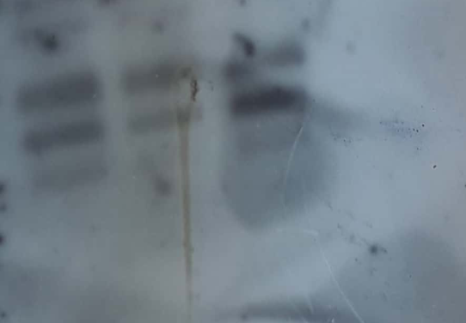


**8C**

**Figure 8:** **Melatonin successfully rectified insulin resistance and enhance signaling proteins in in the young and juvenile offspring brains.**

Shown are the Western blot results of (A) insulin resistance makers (p-IRS, p-Akt and p-GSK3β) and (B) signaling proteins (SIRT1, Nrf-2 and HO-1) in juvenile offspring. Also Shown are the Western blot results of signaling proteins (SIRT1, Nrf-2 and HO-1) in the young offspring brains of the HFD group.
