# Supplementary material for: Melatonin rescues pregnant female mice and their juvenile offspring from high fat diet-induced alzheimer disease neuropathy
Source: Heliyon. 2024 Aug 24;10(17):e36921. doi: 10.1016/j.heliyon.2024.e36921 (PMC11395765; doi:10.1016/j.heliyon.2024.e36921)

**Supplementary materials and method**

**Chemicals and reagents**

Melatonin, sodium dodecyl sulphate (SDS), phosphate buffer saline (PBS), ammonium per sulphate (APS), acrylamide, bis-acrylamide, trizma base, potassium chloride (KCl) and sodium chloride (NaCl) were procured from Sigma Aldrich Chemical Co. (St. Louis, MO, USA) & Daejung Chemicals & Metals Co. Ltd (Gyeonggi-do, Shiheung, South Korea).

## Antioxidant analysis

### Catalase (CAT) assay

CAT activity was estimated by previously developed method with certain modifications [1]. A volume of 0.1 mL of brain supernatant was added to 0.4 mL of 5.9 mM H_2_O_2_ and 2.5 mL of 50 mM phosphate buffer at pH 5.0 to form 3 mL of reaction mixture. Absorbance change of the reaction mixture was taken at 240 nm at one minute interval. Absorbance change of 0.01units/minute was considered as one unit of catalase activity.

### Peroxidase (POD) assay

Slight changes were adapted in the method of Chance and Maehly (1955) to measure peroxidase activity [1]. Hippocampal homogenate supernatant (1 mL) was added to 0.1 mL of 20 mM guaiacol, 0.3 ml of 40 mM H_2_O_2_ and 2.5 mL of 50 mM phosphate buffer at pH 5.0 to form the reaction mixture. Absorbance change of the reaction mixture was taken at 470 nm at one minute interval. Absorbance change of 0.01units/minute was considered as one unit of peroxidase activity.

### Superoxide dismutase (SOD) assay

A total of 0.3 mL hippocampal homogenate supernatant was added to 0.1 mL of 186µM phenazine methosulphate and 1.2 mL of 52 µM sodium pyrophosphate buffer at pH 7.0 to constitute a reaction mixture for the estimation of superoxide dismutase activity. Enzymatic reaction was initiated by the addition of 0.2 mL of 780 µM NADH to the reaction mixture. After 1 minute, 1 mL of glacial acetic acid was added as the stopping agent. Absorbance of reaction mixture was taken at 560nm to determine the amount of chromogen formed. Results were expressed as units per mg of protein [2].

### Glutathione peroxidase (GSH) assay

A total of 1 mL of 4% sulfosalicylic acid was added to an equal volume of hippocampal homogenate for the estimation of glutathione peroxidase levels. Reaction mixture contained 2.7 mL of 0.1 M phosphate buffer at pH 7.4, 0.1 mL of centrifuged aliquot and 0.2 ml of DTNB (100mM). Reaction mixture was centrifuged at 4ºC for 20 min and 1200g after incubation for 1 hour. Absorbance of reaction mixture was taken immediately at 412 nm to determine the amount of glutathione peroxidase. Results were expressed as µM/g tissue [3].

**Estimation of lipid peroxidation (TBARS)**

Estimation of TBARS was performed according to the previously suggested method with slight modifications [4]. Briefly, 0.02 mL of 100 mM ferric chloride, 0.2 mL of 100 mM ascorbic acid and 0.58 mL of 0.1 M phosphate buffer was added to 0.2 mL of hippocampal homogenate supernatant at pH 7.4 to constitute 1 ml reaction mixture. A solution of 1 ml of trichloroacetic acid (10%) was added to the reaction mixture to stop the reaction after 1 hour of incubation in shaking water bath at 37ºC. Tubes were placed in hot water bath (95ºC) for 20 minutes after the addition of 1 ml of 0.67% thiobarbituric acid. Tubes were then rapidly shifted to the ice bath before centrifugation at 2500 g for 10 minutes. Absorbance of supernatant was measured at 535 nm on spectrophotometer to quantify the lipid peroxidation (TBARS) formed in every samples. The results were expressed as nM TBARS/min/mg of tissue at 37ºC (TBARS molar extinction coefficient is 1.56 ×105 M-1cm-1).

**Supplementary Results**

**HFD feeding induces multiple complications in the dams in 1^st^ intervention**

After feeding the dams with HFD for three weeks, the animals showed significant changes in body weight accompanied by deranged lipid profiles and oxidative stress. Significant increase (p<0.001) was observed in the body weight of the female mice (Figure S-3A). HFD induced OS by increasing the burden of ROS. Different antioxidant enzyme assays namely, SOD, POD, GSH, CAT, and LPO (TBARS) were performed with the hippocampal homogenates of experimental dams exposed to normal chow and HFD. These parameters were quantified through Elisa technique. Significant decrease (p<0.001) in CAT levels along with significant increase (p<0.001) in the pro-oxidant LPO levels were observed in the brain homogenate of HFD fed female mice as compared to normal chow diet group. Likewise, Significant decrease (p<0.01) was also evident in the SOD, POD, and GSH levels (Figure S-3B to 3F). Similarly, HFD feeding significantly increased (p<0.001) TC, TG and LDL along with significant increase (p<0.05) in VLDL. HFD feeding significantly decreased (p<0.05) HDL level in the hippocampal homogenates of the dams (Figure S-3G to 3K).

Behavioral assessment revealed that HFD induced both short term and long-term memory impairments. The mean escape latency in the MWM test was significantly high (p<0.001) in HFD group (Figure S-4A). The probe test of MWM revealed that HFD female mice spent significantly little/short time (p<0.01) in the target quadrant as compared to normal chow fed mice (Figure S-4B). Similarly, Y-maze test showed that percentage of spontaneous alternation was significantly less (p<0.001) in HFD group (Figure S-4C).

**Melatonin manifested remarkable amelioration of the dams’ weight**

Dams were weighed twice during the course of the study, once while intervening for base line data before pregnancy and then after parturition. Mice were randomly selected from all the groups i.e. (a) normal chow diet group (b) HFD group and (c) HFD + Mel group for the therapeutic assessment of melatonin against HFD induced weight gain. Significant increase (p<0.001) was observed in the weight of dams fed on HFD as compared to the mice fed on normal chow diet. Melatonin treatment significantly decreased (p<0.001) the weight of HFD fed mice as shown in Figure S-5.

**Melatonin demonstrated substantial reduction in the HFD-induced oxidative stress markers**

Melatonin treatment during pregnancy considerably rectified the OS induced by the HFD in the brains of the dams. One way ANOVA showed significant differences (p<0.001) in the anti-oxidants / pro-oxidant values among all the experimental groups. Post hot Tukey test revealed that brain homogenates of the female mice receiving melatonin displayed highly significant increase (p<0.001) in the POD level. Significant increase (p<0.01) in the GSH level along with significant increase (p<0.05) in the SOD level was observed. Melatonin also caused significant decrease (p<0.01) in the LPO level along with non-significant retrieval of CAT level in post-parturition intervention (Figure S-6 A to E). Melatonin, however significantly rectified (p<0.01) almost all the parameters in the post weaning intervention (S-6 F to J).

Therapeutic efficacy of melatonin on the anti-oxidant enzymes level was also assessed in the mice offspring. One way ANOVA showed significant differences (p<0.01) in the POD, SOD, and CAT levels, while significant differences (p<0.05) were observed in GSH and LPO levels among all the experimental young offspring groups. Post hot tukey test revealed significant decrease (p<0.05) in SOD, (p<0.01) in CAT level, (p<0.001) in POD level along with non-significant change in CAT level in the young offspring hippocampal homogenates. Melatonin also brought significant decrease (p<0.01) in the LPO level in the brain homogenates of the HFD group young offspring brain (Figure S-7 A to E). Similarly, Maternal melatonin treatment significantly rectified (p<0.01) SOD, CAT, and LPO levels in HFD + Mel group juvenile offsprings with non-significant effect on POD and GSH levels in the HFD group adolescent mice brain (Figure S-7 F to J).

**Melatonin exhibited significant rectification in the diabetic status and serum biochemical parameters in all the maternal and offspring interventions**

HFD induced hyperglycemia and altered lipid profile were analyzed in the dams after feeding on HFD, whereas melatonin lowered glucose levels and rectified the changes in lipid profiles in the maternal blood. two way ANOVA showed significant differences (p<0.001) in the IPGTT in both maternal and adult offspring interventions, followed by post hoc Tukey test which showed significant increase (p<0.001) in the IPGTT and RBS levels in the HFD group as compared to normal chow diet group. Melatonin, however markedly retrieved (p<0.001) IPGTT and RBS levels in both maternal interventions (Figure S-8A) and juvenile offspring intervention (Figure S-8B).

Likewise, one-way ANOVA showed significant differences (p<0.001) in all the lipid parameters in both the maternal interventions. Post hoc Tukey test revealed significant increase (p<0.01) in all the lipid parameter in HFD group as compared to normal chow diet group. Melatonin, however significantly rectified (p<0.01) TC, TG and LDL and (p<0.01) HDL and VLDL levels in HFD + Mel groups in both maternal interventions (Figure S-9).

Lipid profile was also analyzed in adult offsprings as well and substantial changes were also observed in various lipids profiles of all groups of adult offsprings. One way ANOVA showed significant differences (p<0.001) in TC, TG, LDL, and VLDL along with Significant decrease (p<0.05) in HDL levels. Post hoc Tukey test demonstrated significant increase (p<0.001) in total TC, TG, LDL, VLDL with significant decrease (p<0.05) in HDL levels in HFD group as compared to normal chow diet. Melatonin significantly rectified (p<0.001) total TC, TG, and LDL, with non-significant effect on HDL and VLDL levels in HFD + Mel group (Figure S-10).

**Melatonin significant rectified the altered behavior in all the maternal and offspring interventions**

Behavioral assessment revealed that HFD induced both short term and long-term memory impairments. Significant differences (p<0.001) were observed in the mean escape latency in the MWM, Probe and Y-maze among various groups of both the maternal interventions. Post hoc comparison showed that the mean escape latency in the MWM was significantly high (p<0.001) in HFD group as compared to the normal chow diet group in both maternal interventions. Like-wise, HFD group mice spent significantly little/short time (p<0.01) in the target quadrant in the probe test. Similarly, Y-maze showed HFD induced memory impairment as the percentage of spontaneous alteration was significantly less (p<0.001) in HFD group in both post parturition and post weaning interventions. Melatonin treatment showed significant retrieval of the damages caused by HFD to both short- and long-term memories of the dams. Significant decrease (p<0.001) in the mean escape latency of the MWM test was observed in both maternal interventions (Figure S-11A and S-11B). Likewise, in the probe test, melatonin successfully increased the time spent in the target quadrant (p<0.05) in the HFD+Mel group in the post-parturition intervention (Figure S-11C) along with significant increase (p<0.001) in the post weaning intervention (Figure S-11D). Similarly, melatonin significantly increased (p<0.001) the percentage of spontaneous alternation in Y-maze test both in both maternal interventions (Figure S-11E, S-11F).

Similarly, behavioral assessment revealed that HFD induced both short term and long-term memory impairments in the juvenile offspring as well**.** Significant differences (p<0.001) were observed in the MWM, Probe test and Y-maze among various groups. Post hoc tukey comparison showed that mean escape latency in the MWM test was significantly high (p<0.001) in HFD group juvenile offspring. Likewise, HFD group adult mice spent significantly little/short time (p<0.001) in the target quadrant and the percentage of spontaneous alternation was significantly less (p<0.001) in Y-maze in HFD group adult mice as compared to normal chow diet group. Melatonin treatment showed remarkable amelioration of the damages caused by HFD to the short and long-term memory of the juvenile offspring. Significant decrease (p<0.01) in the mean escape latency was observed in the MWM test (Figure S-12A). In the probe test of MWM, melatonin significantly increased (p<0.01) the time spent in the target quadrant (Figure S-12B). Similarly, a significant increase (p<0.001) in the percentage of spontaneous alternation in Y-maze test was observed in HFD + Mel group juvenile offspring (Figure S-13C).

**Reference:**

[1] B. Chance, A.C. Maehly, [136] Assay of catalases and peroxidases, (1955).

[2] Y.I. Sun, L.W. Oberley, Y. Li, A simple method for clinical assay of superoxide dismutase., Clin. Chem. 34 (1988) 497–500.

[3] J.B. Owen, D.A. Butterfield, Measurement of oxidized/reduced glutathione ratio, in: Protein Misfolding Cell. Stress Dis. Aging, Springer, 2010: pp. 269–277.

[4] C. Sanmartín-Suárez, R. Soto-Otero, I. Sánchez-Sellero, E. Méndez-Álvarez, Antioxidant properties of dimethyl sulfoxide and its viability as a solvent in the evaluation of neuroprotective antioxidants, J. Pharmacol. Toxicol. Methods. 63 (2011) 209–215.

**Supplementary Figures with legends**

**Figure S-1: Project execution plan**

**Figure S-2: Schematic representation of the animal distribution and experimental interventions.**

**Figure S-3: HFD induced weight gain, oxidative stress and hyperlipidemia in the dams.**

Given are the (A) weight changes, different antioxidants / pro-oxidant enzyme assays (B) POD, (C) SOD, (D) GSH, (E) CAT, and (F) LPO (TBARS) performed with the brain homogenates of experimental dams exposed to normal chow and HFD respectively. These assays were replicated three times. Also given are the bar charts representing serum lipid profiles including (G) Cholesterol, (H) TG, (I) HDL, (J) LDL, (K) VLDL of the 1^st^ maternal intervention. The detailed treatment is given in material and methods. Significance: ^##^p≤0.01 and ^###^p≤0.001. The results are expressed as Mean ± SEM of (n=3) mice in each group.

**Figure S-4: HFD significantly** **altered the behavior of the dams.**

Shown are the memory tests results as (A) Mean escape latency in MWM, (B) Probe test, and (C) percentage of spontaneous alteration in Y-Maze test in pre-pregnancy stage. Significance of control vs HFD is expressed as #. Significance: ##p<0.01 and ###p<0.001.

**Figure S-5:** **Melatonin successfully decreased weight in HFD fed dams.**

Given are the bar charts representing weight changes of the dams. The results are expressed as Mean ± SEM of (n=3) mice each group. Significance of one-way ANOVA is expressed as α, significance of control vs HFD is expressed as #, while significance of HFD vs HFD+Mel is expressed as*. Significance: ***, ###p≤0.001.

**Figure S-6:** **Melatonin demonstrated substantial reduction in the HFD-induced oxidative stress in the post-partum and post-weaning dams’** **brains.**

Given are the different antioxidants / pro-oxidant enzyme assays (A) POD, (B) SOD, (C) GSH, (D) CAT, and (E) LPO (TBARS) performed with the hippocampal homogenates of all the experimental post-partum dams’ groups. Also given are the different antioxidants / pro-oxidant enzyme assays (F) POD, (G) SOD, (H) GSH, (I) CAT, and (J) LPO (TBARS) performed with the hippocampal homogenates of all the experimental post-weaning dams groups. These assays were replicated three times. The detailed treatment is given in material and methods. The results are expressed as Mean ± SEM of (n=3) mice each group. Significance of one-way ANOVA is expressed as ^α^, significance of control vs HFD is expressed as #, while significance of HFD vs HFD+Mel is expressed as*. Significance: ^ααα, ***, ###^p<0.001.

**Figure S-7:** **Melatonin demonstrated substantial reduction in the HFD-induced oxidative stress in the young and juvenile offspring** **brains.**

Given are the different antioxidants / pro-oxidant enzyme assays (A) POD, (B) SOD, (C) GSH, (D) CAT, and (E) LPO (TBARS) performed with the hippocampal homogenates of all the experimental neonatal mice groups. Also given are the different antioxidants / pro-oxidant enzyme assays (F) POD, (G) SOD, (H) GSH, (I) CAT, and (J) LPO (TBARS) performed with the hippocampal homogenates of all the experimental adolescent mice groups. These assays were replicated three times. The detailed treatment is given in material and methods. The results are expressed as Mean ± SEM of (n=3) mice each group. Significance of one-way ANOVA is expressed as ^α^, significance of control vs HFD is expressed as #, while significance of HFD vs HFD+Mel is expressed as*. Significance: ^ααα, ***, ###^p<0.001.

**Figure S-8:** **Melatonin showed immense amelioration of the hyperglycemia in both the maternal interventions.**

Given are the bar charts representing (A) IPGTT (post-partum females), (B) IPGTT (post-weaning females), (C) RBS levels (post-partum females) and (D) RBS levels (post-weaning females) of all the three experimental groups. The detailed treatment is given in material and methods. The results are expressed as Mean ± SEM of (n=3) mice each group. Significance of two-way ANOVA (IPGTT) and one way ANOVA (RBS) is expressed as ^α^, significance of control vs HFD is expressed as #, while significance of HFD vs HFD+Mel is expressed as*. Significance: ^ααα, ***, ###^p<0.001.

**Figure S-9:** **Melatonin manifested remarkable amelioration of the hyperlipidemia in the dams.**

Given are the bar charts representing serum lipid profiles including (A) TC (B) TG, (C) HDL, (D) LDL, and (E) VLDL of the post-partum dams. Also given are the bar charts representing serum lipid profiles including (F) TC (G) TG, (H) HDL, (I) LDL, (J) VLDL of post-weaning female mice of all the experimental groups. These assays were replicated three times. The detailed treatment is given in material and methods. The results are expressed as Mean ± SEM of (n=3) mice each group. Significance of one-way ANOVA is expressed as ^α^, significance of control vs HFD is expressed as #, while significance of HFD vs HFD+Mel is expressed as*. Significance: ^ααα, ***, ###^p<0.001.

**Figure S-10:** **Melatonin showed immense amelioration of the hyperglycemia and hyperlipidemia in juvenile mice.**

Given are the bar charts representing (A) IPGTT, (B) RBS levels in adolescent mice. Also given are the bar charts representing serum lipid profiles including (A) TC (B) TG, (C) HDL, (D) LDL, and (E) VLDL of the juvenile mice of all the three experimental groups. These assays were replicated three times. The detailed treatment is given in material and methods. The results are expressed as Mean ± SEM of (n=3) mice each group. Significance level of IPGTT is evaluated by two-way ANOVA. Significance of one-way ANOVA is expressed as ^α^, significance of control vs HFD is expressed as #, while significance of HFD vs HFD+Mel is expressed as*. Significance: ^ααα, ***, ###^p<0.001.

**Figure S-11: Melatonin manifested remarkable amelioration in the behaviour of HFD fed dams.**

Given are the memory tests results as Mean escape latency in MWM (A) in post-parturition stage and (B) in post-weaning stage, Probe test (C) in post-parturition stage and (D) in post-weaning stage and percentage of spontaneous alteration in Y-Maze test (E) in post-parturition stage and (F) in post-weaning stage. The treatment details have already been given in material and methods. Significance of two-way ANOVA (MWM) and one way ANOVA (Probe test and Y-Maze) is expressed as α, significance of control vs HFD is expressed as #, while significance of HFD vs HFD+Mel is expressed as*. Significance: αα, **, ##p<0.01 and ααα, ***, ###p<0.001.

**Figure S-12: Melatonin significantly rectified the altered behavior of the juvenile mice**

Given are the memory tests results as mean escape latency in MWM, (C) Probe test, (C) and percentage of spontaneous alteration in Y-Maze test. The treatment details have already been given in material and methods. Significance of two-way ANOVA (MWM) and one way ANOVA (Probe test and Y-Maze) is expressed as α, significance of control vs HFD is expressed as #, while significance of HFD vs HFD+Melationin is expressed as*. Significance: αα, **, ##p<0.01 and ααα, ***, ###p<0.001.

**Figure S-1**


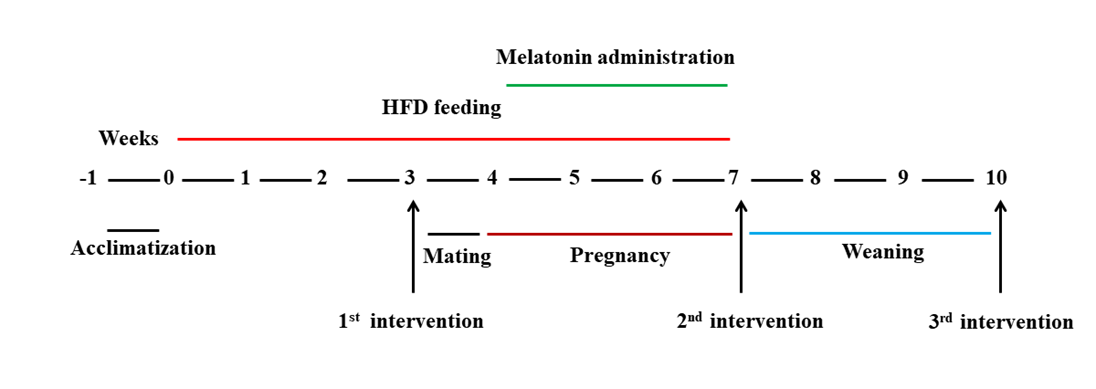


**Figure S-2:**


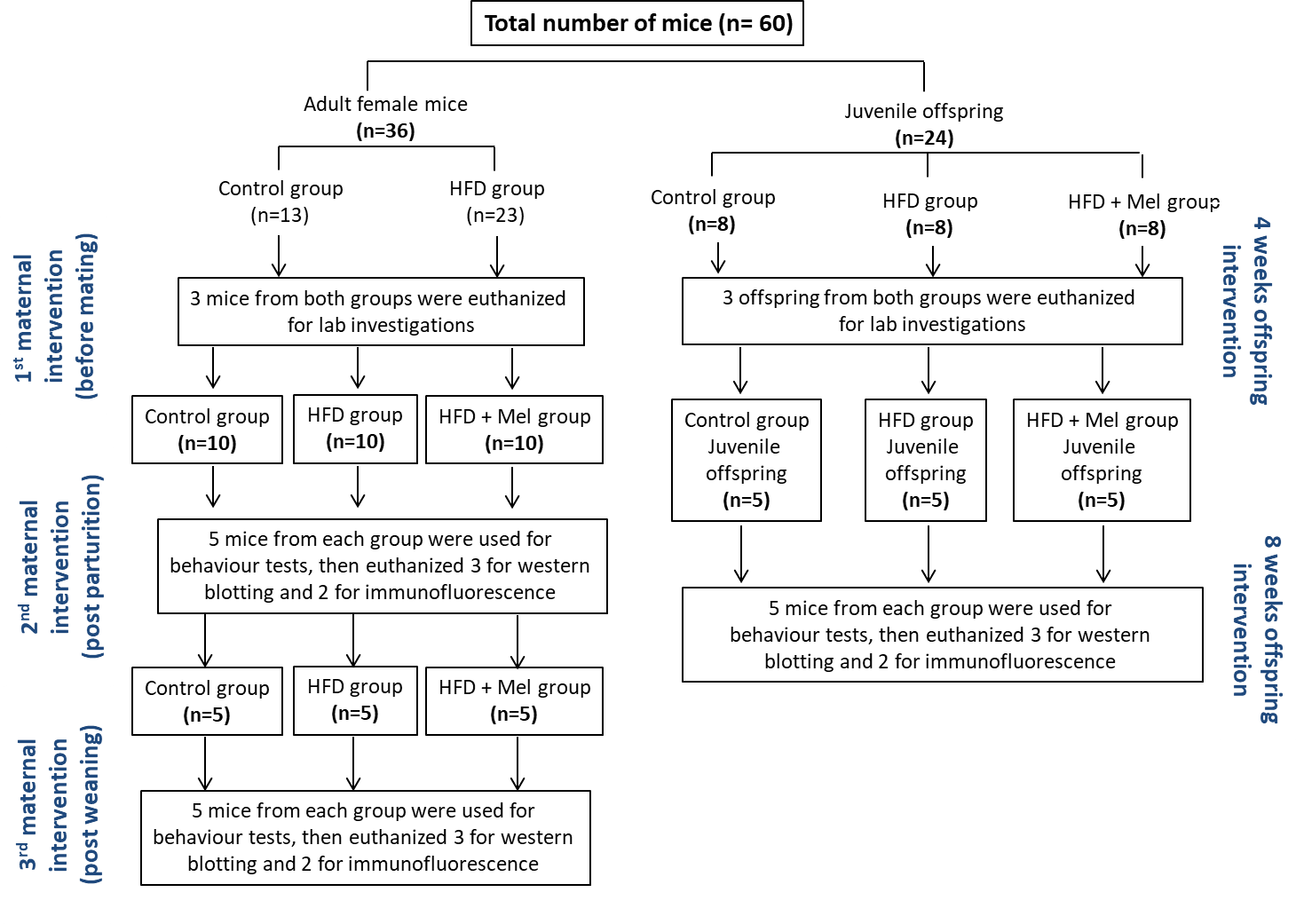


**Figure S-3:**

**
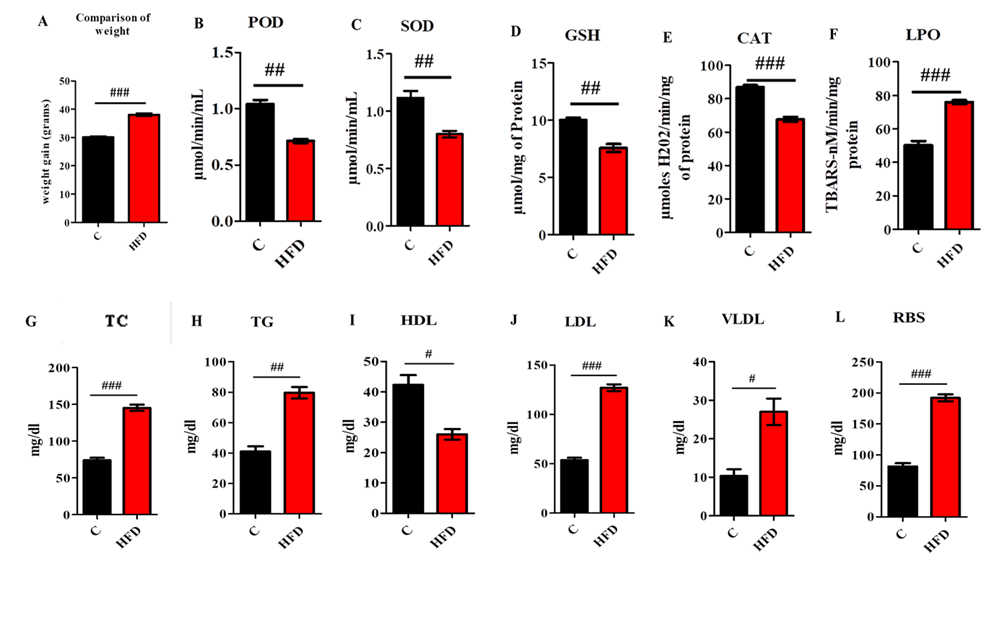
**

**Figure S-4:**


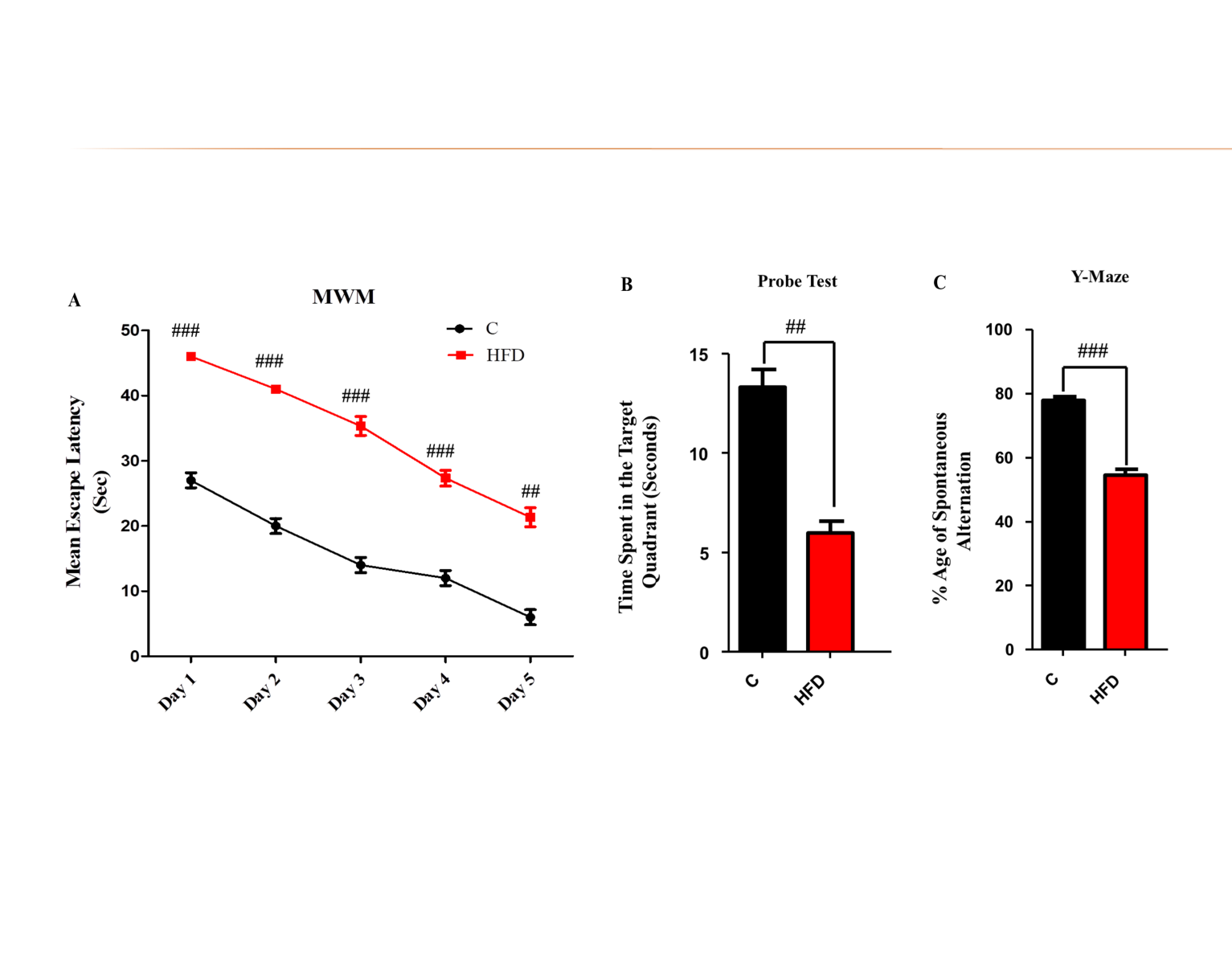


**Figure S-5:**


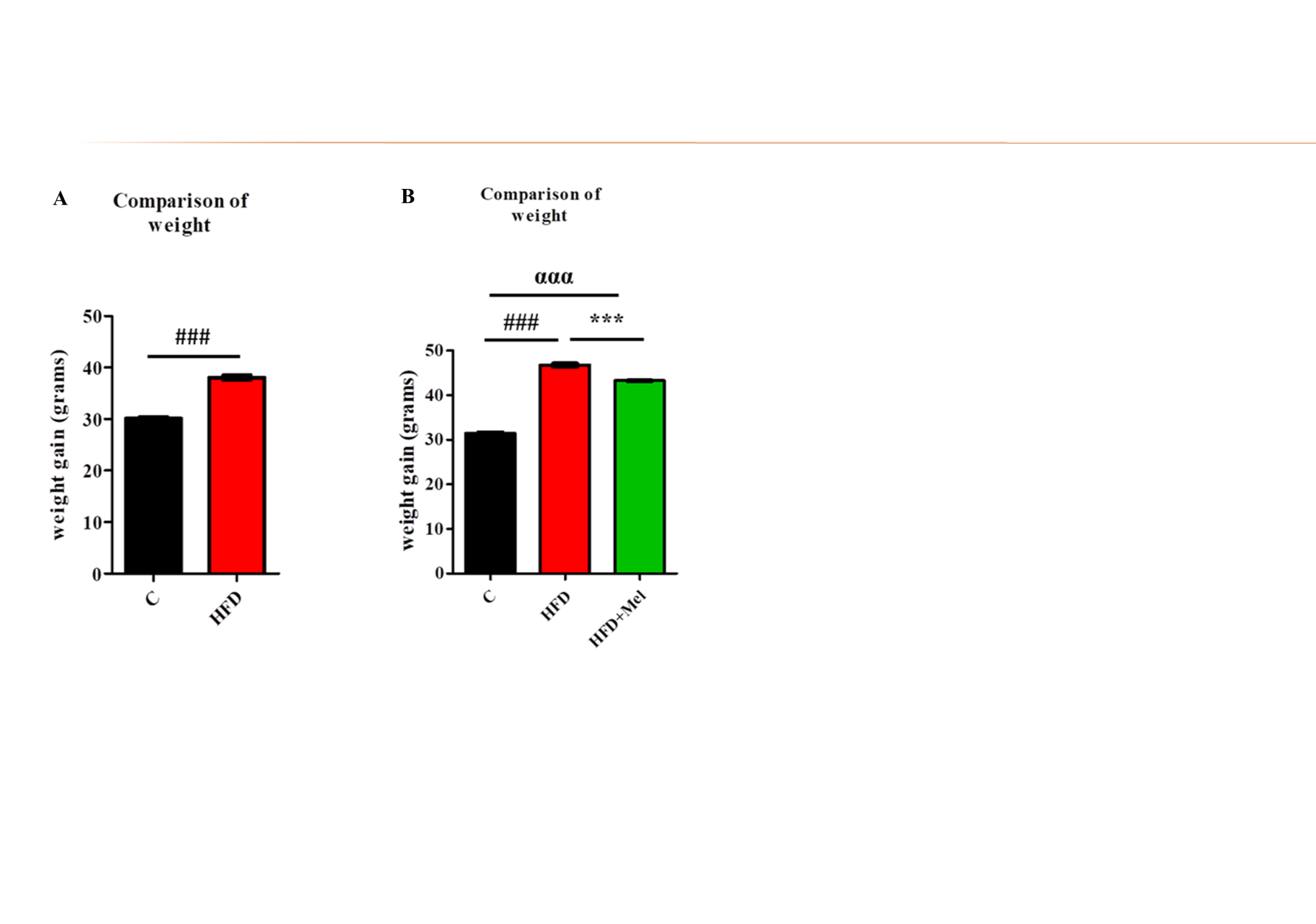


**Figure S-6:**

**
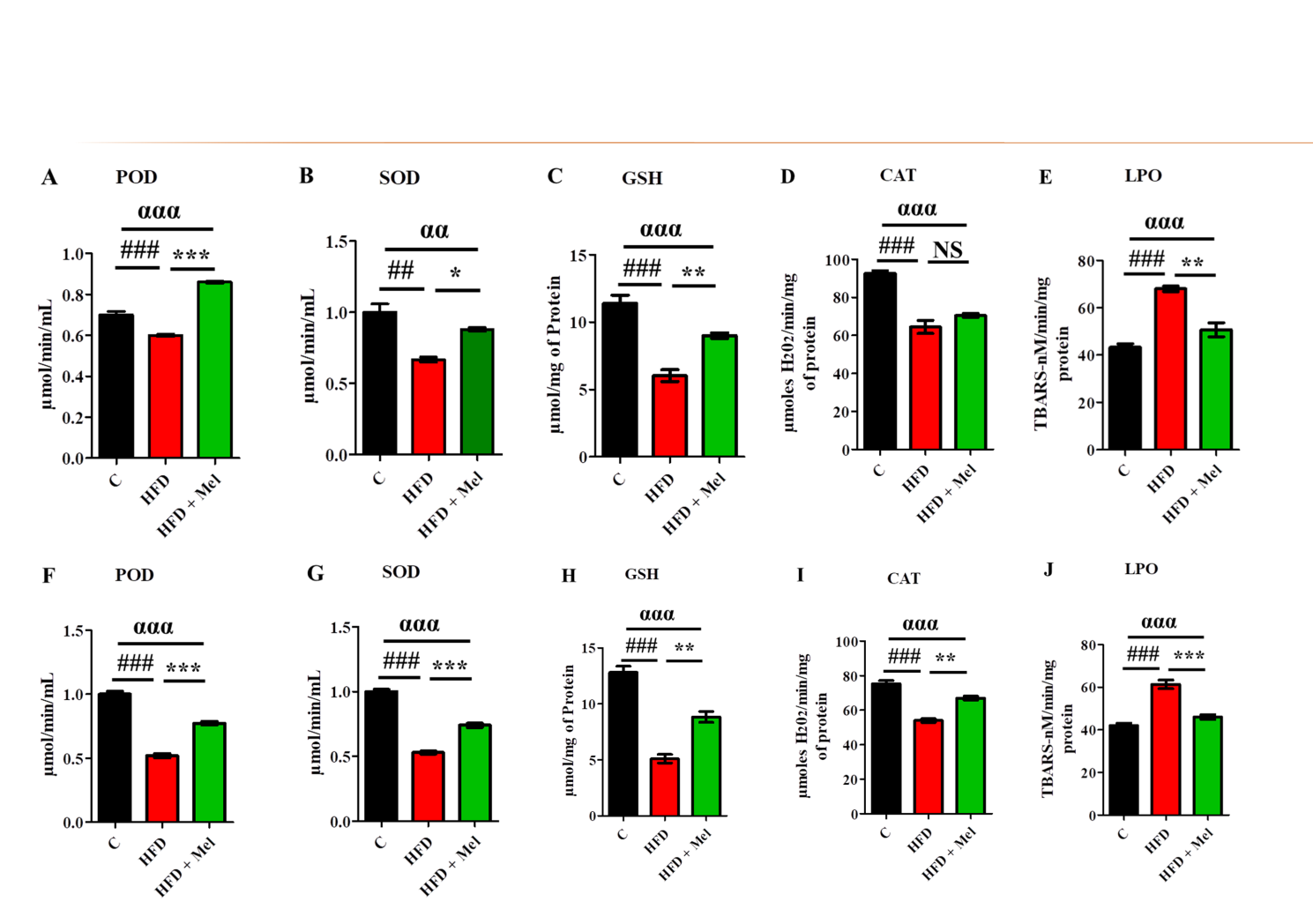
**

**Figure S-7:**

**
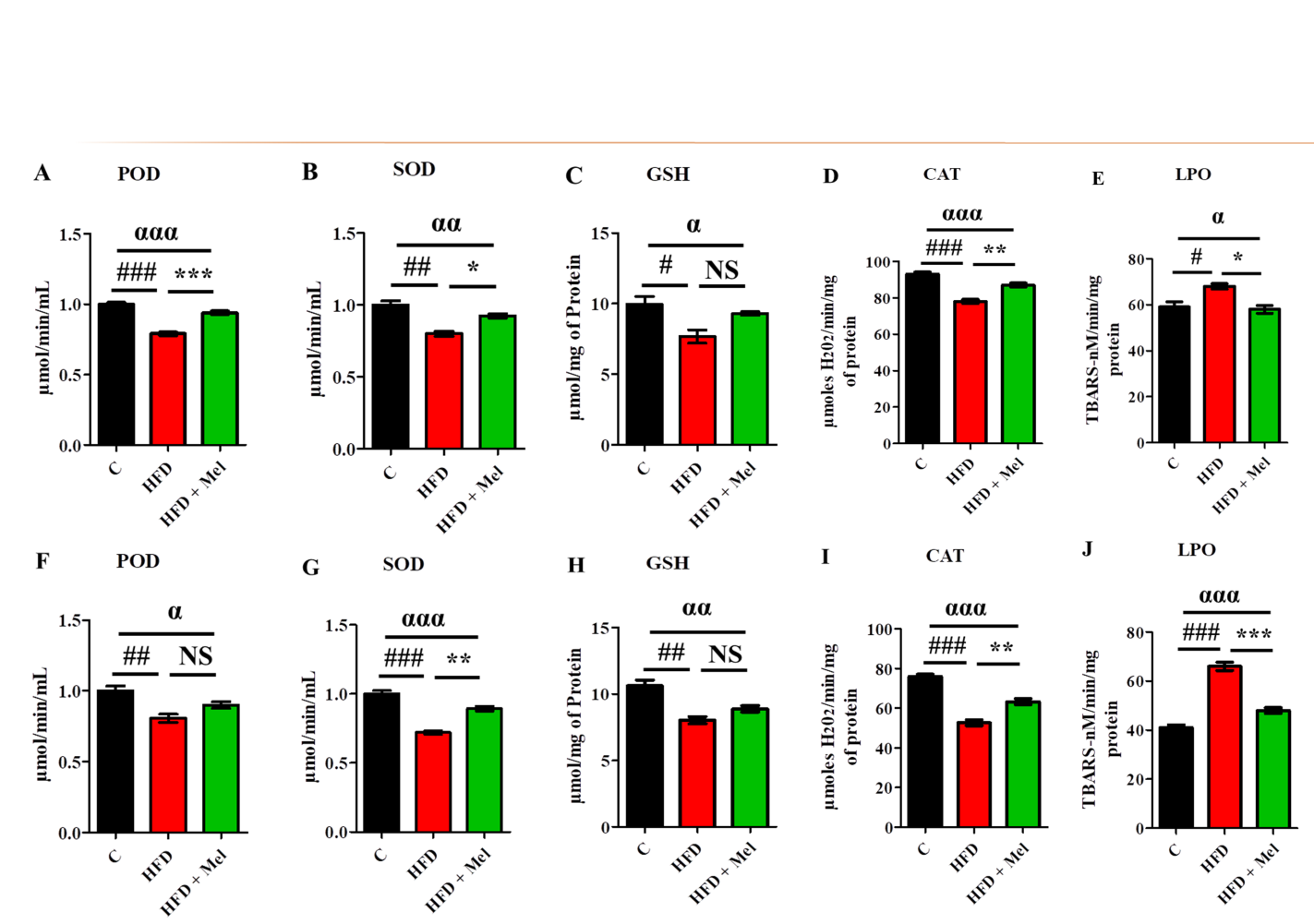
**

**Figure S-8:**

**
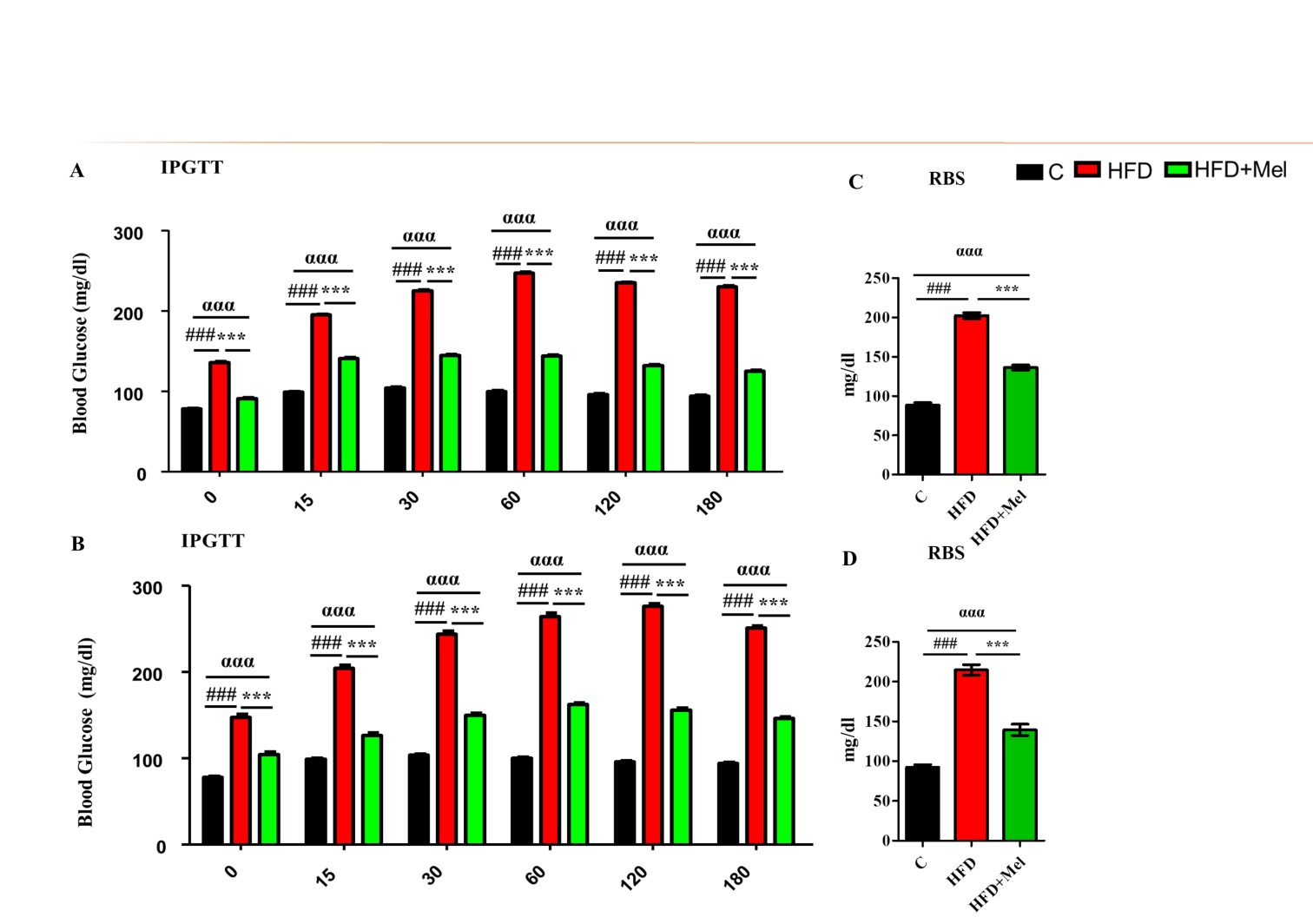
**

**Figure S-9:**


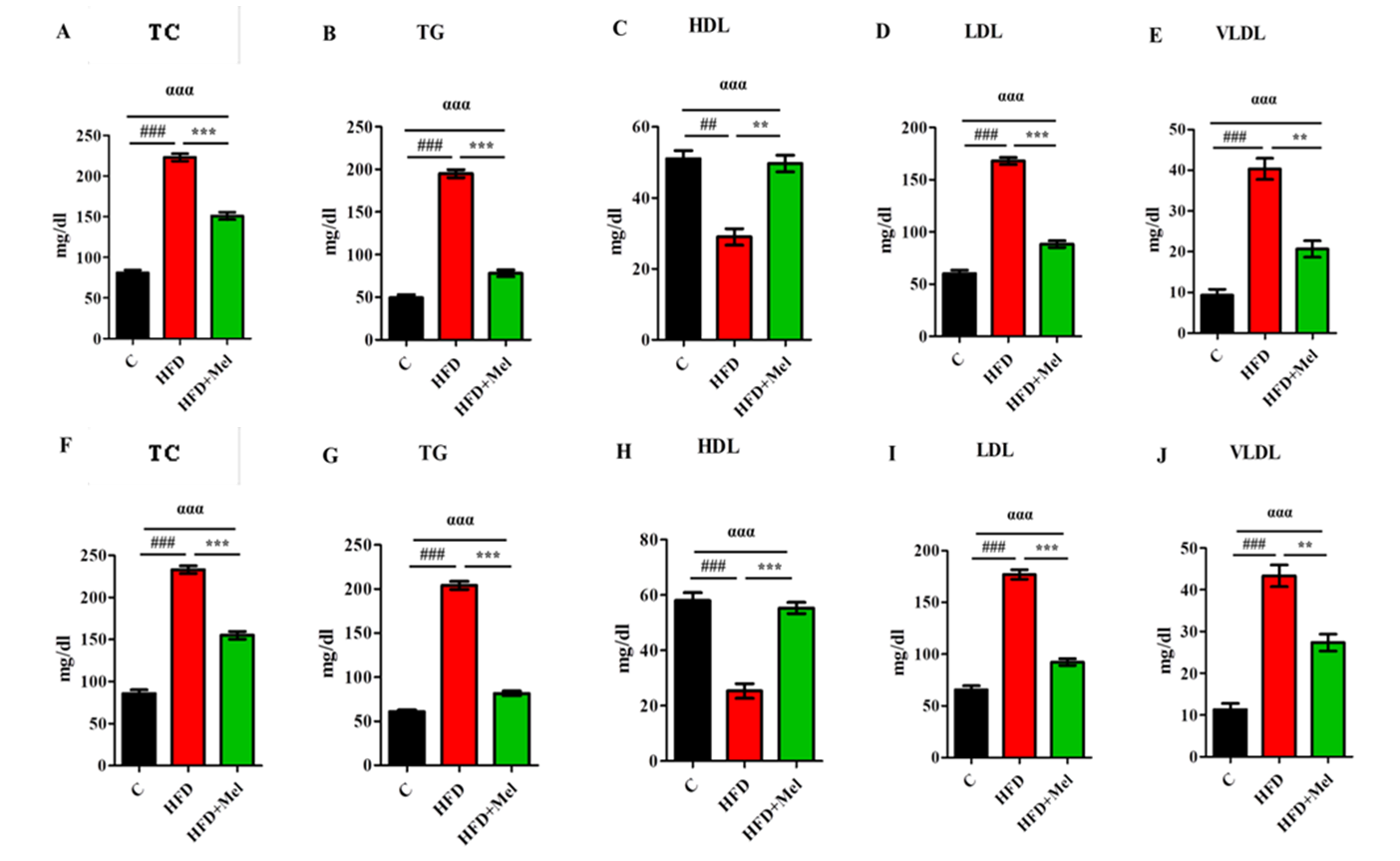


**Figure S-10:**


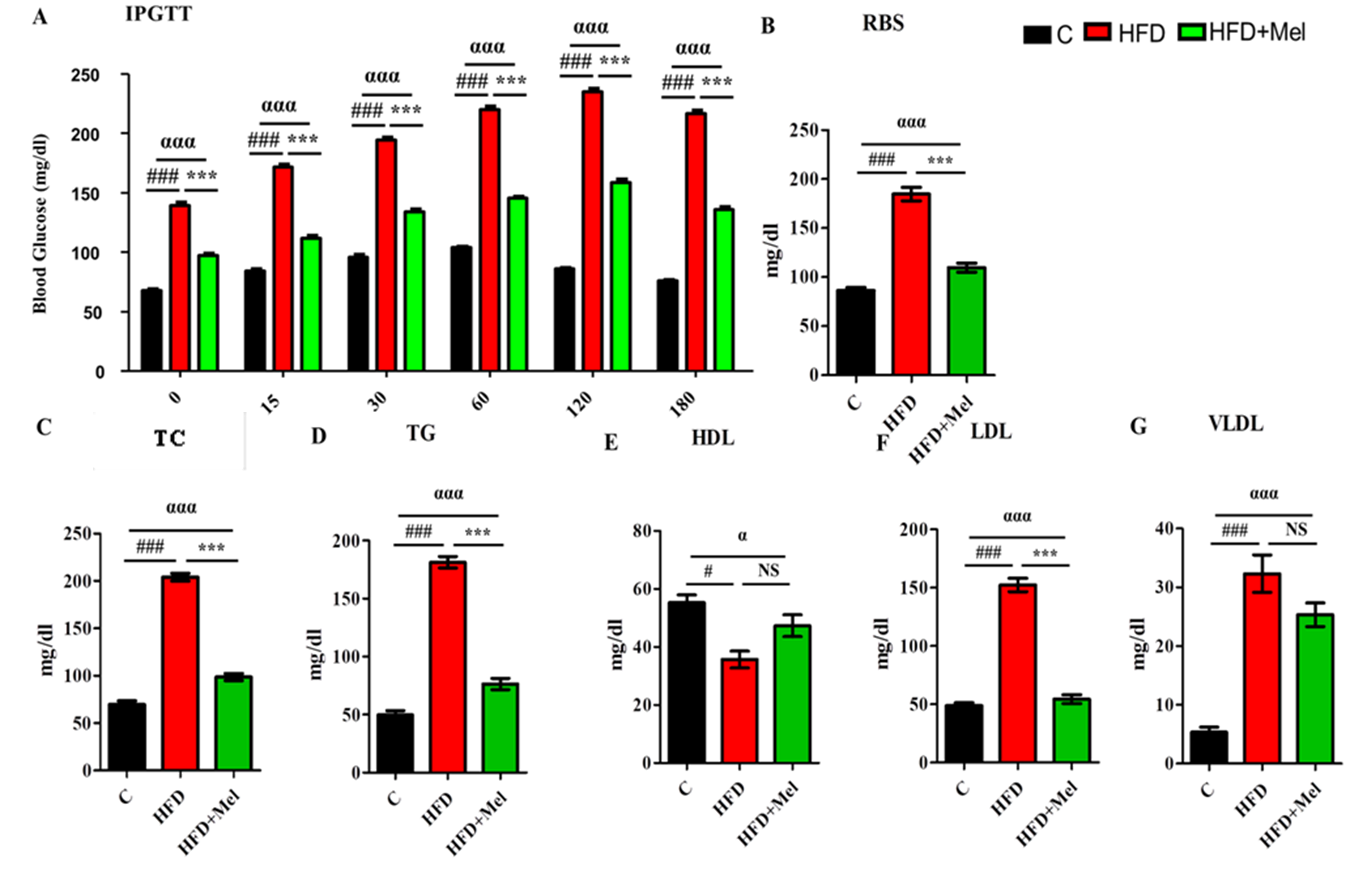


**Figure S-11:**

**
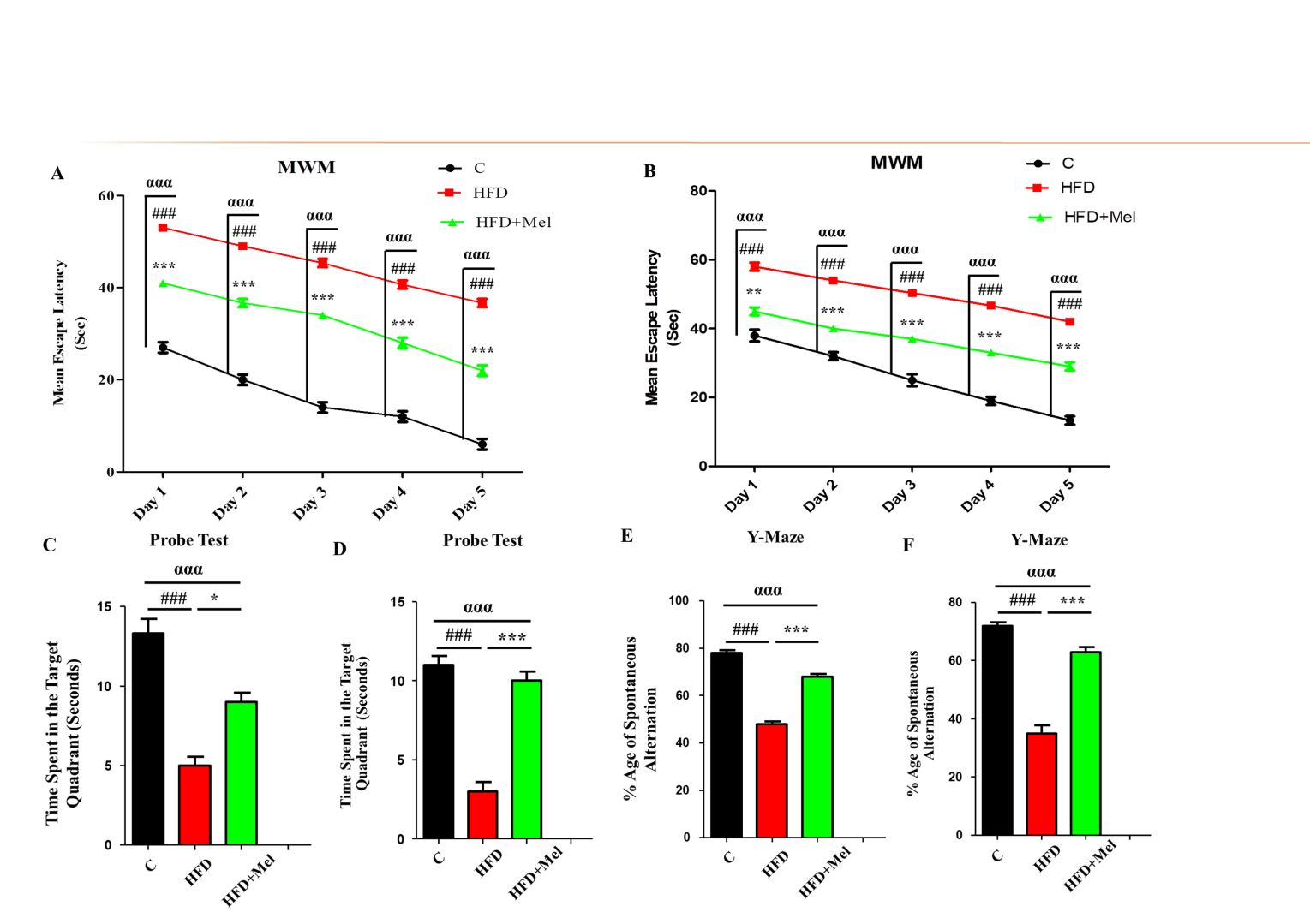
**

**Figure S-12:**


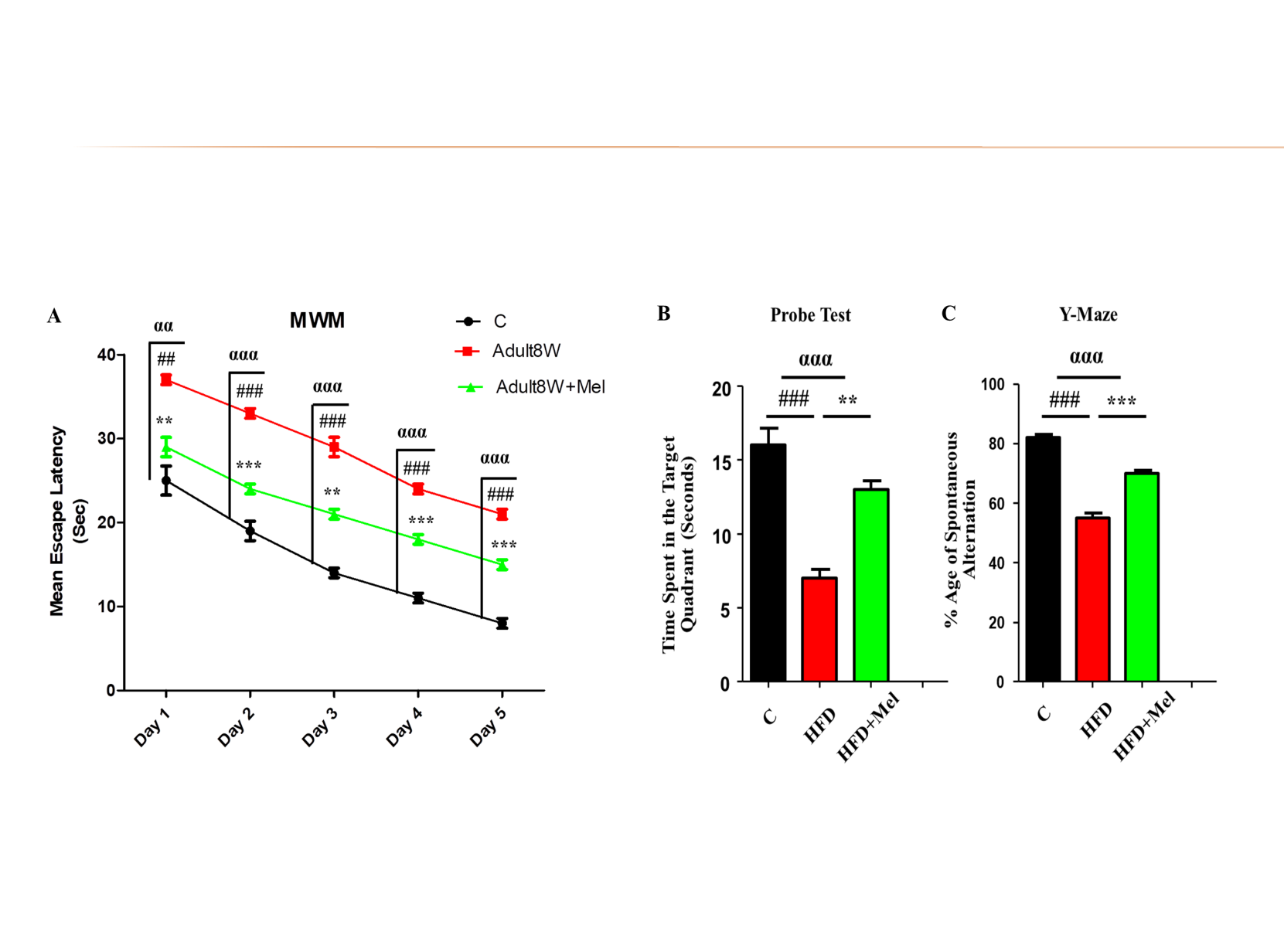

Supplement: Multimedia component 2 [file mmc2.docx]
